# Supplementary material for: COVID-19 mortality risk assessment: An international multi-center study
Source: PLoS One. 2020 Dec 9;15(12):e0243262. doi: 10.1371/journal.pone.0243262 (PMC7725386; doi:10.1371/journal.pone.0243262)
Supplement: S1 File — (PDF) [file pone.0243262.s001.pdf]

# S1 File

## S1 Text. Population characteristics.

The clinical features of each derivation and validation cohort are described in S1 and S2 Tables. We observe differing rates of comorbidities in the populations: our identification of comorbidities was limited by how they were captured in an admission's diagnosis list. Chronic conditions that did not appear in the diagnosis list are considered to not be present in the patient, which could lead to under-reporting of comorbidities. While this is a limitation, these features are not significant in the model on their own and thus do not greatly affect the model predictions. As we expand our models to incorporate richer medical history and treatment information, we will revisit this topic.

## S2 Text. Missing data imputation.

Missing values were encountered in the majority of the included risk factors since the electronic health records of many patients were not complete. Employing imputation techniques instead of complete case analysis allows the inclusion of a wider set of features which otherwise would have been omitted by the model. The $k$-Nearest Neighbors algorithm [1] is a machine learning technique that can be applied to both supervised and unsupervised learning problems. In the missing data imputation setting, given a missing value for a patient, the algorithm searches for $k$ observations in the population that are nearest in feature space, where $k = 5$ in our analysis. The observation is then imputed to the average of the values of its neighbors belong. Though the $k$-NN algorithm is a simple technique, it often has powerful empirical performance. Its simplicity is also an advantage in terms of interpretability – one can assess the imputed value of a certain point by looking at its neighbors and in which features they are most similar. The training set was imputed independently of the testing set to avoid any bias in the resulting data.

## S3 Text. The XGBoost algorithm.

The XGBoost algorithm is one of the most popular ensemble methods for binary classification in the machine learning field [2]. It is based on a large number of trees that are built in an iterative fashion. Later trees are constructed based on the errors that existed in earlier trees, giving the model more power to handle "harder" cases. This error correction ability often gives XGBoost a performance edge over other linear or tree-based methods. There is multitude of hyperparameters that need to be tuned for this algorithm. Three of them are particularly important: number of trees, depth of trees and learning rate. In this study, we tune the parameters: learning rate, $\gamma$, $\lambda$, $\alpha$, minimum child weight, maximum tree depth, number of estimators. The learning rate, also called shrinkage factor or $\eta$, controls the weighting factor for corrections by new trees added in the model: it takes values between 0 and 1, with values closer to 1 having more corrections for each tree and higher risk of overfitting on the training data. Gamma ($\gamma$) is a regularization parameter controlling the minimum loss reduction required to make a further partition on a leaf node of a tree: it takes positive values, with larger ones defining a more conservative model. Lambda ($\lambda$) is the L2 regularization parameter on the feature weights: it takes positive values, with the larger ones encouraging smaller weights, thus making the model more conservative. Alpha ($\alpha$) is the L1 regularization parameter on the feature weights: it takes positive values, with the larger ones driving to 0 the weights, defining a more conservative model. Minimum child weight is the minimum Hessian weight required to create a new node, with a role similar to that of $\gamma$, i.e. regularization at the splitting step: it takes positive values, with higher values making the model more conservative. The maximum depth of a tree controls the maximum number of nodes that can exist between the root node and the farthest leaf in the tree: it is a positive integer, and large values usually lead to overfitting on the training data. The number of estimators determines the number of trees to fit in the model: it is a positive integer, and large values usually lead to overfitting on the training data. All remaining parameters are set to their default values.

## S4 Text. Methods comparison.

We compared three different machine learning methods in the development of our model. In all cases, we formulate a binary classification problem to predict mortality (1) or discharge (0) as the endpoint of a patient's

hospitalization. Predictive models are trained using XGBoost, Logistic Regression, and Classification And Regression Trees (CART); all methods are implemented in Scikit-learn [3]. Logistic Regression assumes an additive relationship between risk factors, whereas CART and XGBoost are able to capture non-linearities and feature interactions. While CART forms a single decision tree, XGBoost is an ensemble method: it constructs a set of decision trees which are then combined to yield a single prediction for a given patient.

S3 Table reports the AUC and various threshold-based metrics for the three algorithms. For each method, we select the threshold that yields a sensitivity of at least 80% to reflect the priority of correctly identifying mortality. Of the three methods, XGBoost is able to capture the most sophisticated interactions between features and subsequently demonstrates the strongest performance. Logistic Regression reports a strong test set AUC but indicates a loss in specificity and precision for the chosen thresholds. CART has the highest negative predictive value but is outperformed by both other models on all other metrics.

### S5 Text. Parameter tuning.

In this project, we leverage the hyperparameter optimization framework Optuna [4] as follows. We first identify the corresponding parameter spaces for the Scikit-Learn implementations of XGBoost, Logistic Regression and CART [3]. Second, we define the objective function as the 300-folds cross validation area under the curve (AUC). Finally, we employ a pipeline to maximize the objective over 500 maximum iterations on multiple cores.

### S6 Text. SHAP methodology.

SHapley Additive exPlanations (SHAP) are useful tools to interpret model predictions and risk drivers [5, 6]. The SHAP methodology explains a patient risk prediction (normalized between 0 and 1) by computing the contribution of each feature. This is obtained by approximating the nonlinear XGBoost prediction model as a linear model around the patient prediction. The coefficients of the linear approximation are estimated by introducing every feature one at a time and comparing the model output variations. We use the SHAP Python package [6], featuring an efficient algorithm to compute the SHAP values and the plot generation functions, to interpret the outcomes of XGBoost model in Figure 1.

## Tables

**S1 Table. Descriptive summary of derivation population broken down by study site.**

**S2 Table. Descriptive summary of validation population broken down by study site.**

**S3 Table. AUC performance and threshold-based metrics for different machine learning methods, evaluated on the test set from the derivation cohort.**

**S4 Table. Overview of participating institutions in the The Hellenic COVID-19 Study Group.**

## Figures

**S1 Figure. Receiver operator curves (ROC) evaluating the model's performance on the testing set for patient subgroups.**

**S1 Table. Descriptive summary of derivation population broken down by study site.**

| | Cremona (N = 1441) | | HM Hospitals (N = 1390) | | Hartford Affiliates (N = 231) | |
|---|---|---|---|---|---|---|
| | Median (IQR) | Missing % | Median (IQR) | Missing % | Median (IQR) | Missing % |
| Age | 70.0 (58.0-80.0) | 1.60% | 67.0 (56.0-78.0) | 1.60% | 68.0 (55.5-79.0) | 1.70% |
| Female * | 558.0 (38.7%) | 0.00% | 537.0 (38.6%) | 0.00% | 112.0 (48.5%) | 0.00% |
| Heart Rate (bpm) | 89.0 (79.1-100.0) | 11.00% | 90.0 (80.0-102.0) | 8.80% | 99.0 (88.0-110.0) | 4.80% |
| Oxygen Saturation (%) | 93.9 (90.2-96.0) | 36.80% | 94.0 (92.0-96.0) | 10.60% | 93.0 (89.0-95.0) | 4.80% |
| Temperature (°C) | 37.2 (36.6-37.9) | 3.30% | 36.6 (36.2-37.2) | 9.50% | 37.8 (37.0-38.6) | 1.30% |
| ALT (U/L) | 26.0 (17.0-43.0) | 4.90% | 27.8 (17.2-44.7) | 19.60% | 25.0 (16.0-41.0) | 15.20% |
| AST (U/L) | 37.0 (26.0-56.0) | 10.10% | 34.7 (25.0-52.9) | 18.70% | 34.0 (24.0-51.8) | 17.70% |
| Blood Glucose (mg/dL) | 119.0 (106.0-144.0) | 6.20% | 116.0 (104.0-137.5) | 10.60% | 122.0 (102.0-159.5) | 5.20% |
| BUN (mg/dL) | 18.0 (14.0-29.0) | 7.80% | 15.5 (12.0-22.3) | 11.10% | 19.0 (12.0-31.0) | 6.10% |
| CRP (mg/L) | 76.3 (28.5-158.6) | 3.70% | 70.9 (29.0-132.5) | 6.80% | 77.3 (30.4-124.0) | 46.80% |
| Creatinine (mg/dL) | 1.0 (0.8-1.3) | 4.10% | 0.9 (0.7-1.1) | 5.70% | 1.0 (0.8-1.4) | 6.50% |
| Hemoglobin (U/g) | 13.5 (12.4-14.7) | 22.30% | 14.2 (13.1-15.2) | 1.90% | 12.6 (11.2-13.9) | 3.90% |
| MCV ($\mu m^3$) | 87.3 (84.6-90.5) | 23.60% | 88.2 (85.5-91.4) | 1.30% | 90.0 (86.0-94.0) | 6.50% |
| Platelets ($10^3/\mu L$) | 198.0 (154.0-261.5) | 22.90% | 204.5 (159.0-259.2) | 2.40% | 204.0 (162.5-250.0) | 6.90% |
| Potassium (mEq/L) | 3.9 (3.6-4.3) | 9.60% | 4.2 (3.9-4.6) | 6.50% | 4.0 (3.7-4.4) | 6.50% |
| Prothrombin Time (INR) | 1.0 (1.0-1.1) | 22.30% | 1.2 (1.1-1.3) | 29.60% | 1.1 (1.1-1.4) | 67.50% |
| Sodium (mEq/L) | 138.0 (136.0-140.0) | 4.20% | 136.6 (134.6-139.0) | 8.30% | 136.0 (134.0-140.0) | 4.30% |
| WBC (/$\mu$L) | 6900 (5300-9400) | 22.80% | 6600 (5100-8900) | 2.90% | 6500 (4800-8700) | 3.50% |
| Cardiac dysrhythmias * | 60.0 (4.2%) | 0.00% | 140.0 (10.1%) | 0.00% | 1.0 (0.4%) | 0.00% |
| Chronic kidney disease * | 16.0 (1.1%) | 0.00% | 49.0 (3.5%) | 0.00% | 7.0 (3.0%) | 0.00% |
| Heart disease * | 48.0 (3.3%) | 0.00% | 77.0 (5.5%) | 0.00% | 0.0 (0.0%) | 0.00% |
| Diabetes * | 138.0 (9.6%) | 0.00% | 207.0 (14.9%) | 0.00% | 39.0 (16.9%) | 0.00% |
| Mortality * | 472.0 (32.8%) | 0.00% | 239.0 (17.2%) | 0.00% | 49.0 (21.2%) | 0.00% |

* Count (proportion) is reported for binary variables.

**S2 Table. Descriptive summary of validation population broken down by study site.**

| | Hellenic CSG (N = 323) | | Seville (N = 219) | | Hartford Hospital (N = 323) | |
|---|---|---|---|---|---|---|
| | Median (IQR) | Missing % | Median (IQR) | Missing % | Median (IQR) | Missing % |
| Age | 59.0 (47.0-72.0) | 0.31% | 64.0 (54.0-78.5) | 0.00% | 73.0 (57.0-84.0) | 0.00% |
| Female * | 125.0 (38.7%) | 0.00% | 91.0 (41.55%) | 0.00% | 176.0 (54.49%) | 0.00% |
| Heart Rate (bpm) | 88.0 (80.0-98.0) | 4.95% | 88.0 (77.0-100.0) | 37.44% | 98.0 (86.0-112.75) | 0.31% |
| Oxygen Saturation (%) | 95.0 (92.0-97.0) | 16.72% | 95.0 (92.0-97.0) | 8.22% | 93.0 (90.0-95.0) | 0.31% |
| Temperature (°C) | 38.0 (37.2-38.5) | 5.57% | 38.5 (38.0-38.9) | 42.01% | 37.8 (37-38.4) | 0.31% |
| ALT (U/L) | 27.0 (18.0-40.0) | 1.86% | 24.0 (16.5-39.5) | 10.96% | 24.0 (16.0-39.0) | 12.69% |
| AST (U/L) | 29.0 (22.0-41.0) | 0.62% | 28.0 (21.0-39.75) | 11.42% | 38.0 (29.0-58.0) | 11.76% |
| Blood Glucose (mg/dL) | 106.0 (95.0-124.0) | 1.86% | 111.5 (95.0-129.0) | 21.46% | 127.0 (107.0-165.5) | 4.02% |
| BUN (mg/dL) | 24.0 (14.56-33.8) | 1.55% | 16.82 (12.15-24.53) | 21.46% | 20.0 (13.0-33.0) | 4.64% |
| CRP (mg/L) | 53.7 (13.0-130.7) | 1.86% | 66.9 (23.45-138.45) | 7.31% | 67.85 (33.9-129.38) | 35.60% |
| Creatinine (mg/dL) | 0.9 (0.7-1.1) | 1.55% | 0.9 (0.76-1.15) | 0.00% | 1.0 (0.8-1.5) | 4.33% |
| Hemoglobin (U/g) | 13.3 (12.2-14.5) | 3.10% | 13.4 (11.8-14.88) | 2.28% | 12.2 (10.8-13.7) | 3.41% |
| MCV ($\mu m^3$) | 86.9 (83.9-89.9) | 2.48% | 91.1 (88.25-94.18) | 2.28% | 90.0 (86.0-95.0) | 3.72% |
| Platelets ($10^3/\mu L$) | 193.0 (156.0-245.0) | 0.93% | 204.5 (163.75-261.75) | 2.28% | 183.5 (140.0-241.5) | 4.02% |
| Potassium (mEq/L) | 4.1 (3.9-4.4) | 1.24% | 3.9 (3.6-4.3) | 0.91% | 4.1 (3.8-4.5) | 4.95% |
| Prothrombin Time (INR) | 1.03 (0.96-1.11) | 4.95% | 1.08 (1.01-1.2) | 73.52% | 1.2 (1.1-1.4) | 53.87% |
| Sodium (mEq/L) | 138.0 (135.0-140.0) | 1.55% | 139.0 (136.0-141.0) | 0.91% | 138.0 (135.0-140.0) | 4.64% |
| WBC (/$\mu$L) | 5710 (4380-7430) | 1.55% | 7180 (5200-10050) | 2.28% | 6800 (5000-9500) | 3.72% |
| Cardiac dysrhythmias * | 45.0 (13.98%) | 0.31% | nan (nan%) | 100.00% | 0.0 (0.0%) | 0.00% |
| Chronic kidney disease * | 16.0 (4.97%) | 0.31% | 21.0 (9.95%) | 3.65% | 10.0 (3.1%) | 0.00% |
| Heart disease * | 60.0 (18.63%) | 0.31% | 55.0 (25.82%) | 2.74% | 0.0 (0.0%) | 0.00% |
| Diabetes | 42.0 (13.04%) | 0.31% | 32.0 (15.02%) | 2.74% | 61.0 (18.89%) | 0.00% |
| Mortality * | 32.0 (9.91%) | 0.00% | 28.0 (12.79%) | 0.00% | 46.0 (14.24%) | 0.00% |

* Count (proportion) is reported for binary variables.

**S3 Table. AUC performance and threshold-based metrics for different machine learning methods, evaluated on the test set from the derivation cohort.**

| Method | AUC | Threshold | Accuracy | Specificity | Precision | NPV |
|---|---|---|---|---|---|---|
| XGBoost | 90.19 (86.86,93.52) | 28.3 (23.26,33.34) | 85.02 (81.02,89.01) | 86.58 (82.77,90.39) | 66.3 (61.02,71.59) | 93.02 (90.17,95.87) |
| Logistic Regression | 88.45 (84.87,92.02) | 21.99 (17.36,26.62) | 80.46 (76.02,84.89) | 80.52 (76.09,84.95) | 57.55 (52.02,63.08) | 92.54 (89.6,95.48) |
| CART | 85.85 (81.95,89.75) | 23.4 (18.67,28.14) | 79.8 (75.31,84.3) | 77.49 (72.82,82.16) | 55.93 (50.38,61.49) | 94.71 (92.2,97.21) |

**S4 Table. Overview of participating institutions in the The Hellenic COVID-19 Study Group.**

| Organization | Sample Size | Study Dates | Description |
|---|---|---|---|
| Sotiria Thoracic Diseases Hospital of Athens | 83 | 03/12 – 05/07 | The Sotiria Thoracic Diseases Hospital of Athens is a tertiary care hospital and the reference centre for respiratory medicine in Greece with a capacity of 710 inpatient beds, of which 400 are dedicated to pulmonary care and ICU. There is also a large sector of internal medicine and infectious disease. The clinic cares for around 150.000 in- and outpatients yearly and is on emergency rota almost daily admitting patients from the large Athens area (5.000.000 inhabitants). The Hospital was the referral centre for COVID infection in Greece and stopped all other operations and admissions during the pandemic. There were 278 admissions, most patients have been discharged and there were 28 deaths. |
| Evangelismos Hospital | 82 | 03/10 – 05/04 | It is the largest tertiary hospital in Greece. It is a referral center for patients with Covid-19 for ICUs and a secondary center for patients in need of hospitalization. The data comes from the Covid - 19 Patient Care Unit set up in a former 90-bed surgery wards. The staff of the clinic was provided by the internal medicine and pulmonary clinics of the hospital as well as colleagues of other hospitals who were seconded to Evangelismos including specialized internists of the hospital. |
| University Hospital of Alexandroupolis | 50 | 03/14 – 05/10 | It is the COVID-19 Reference Hospital for the Region of Eastern Macedonia - Thrace, an area with a large heterogeneity of population. It includes a total of 572 beds, of which 40 (Special Infections Unit and COVID-19 Clinic) exclusively for patients with SARSCoV-2, as well as the ICU (16 beds). |
| University Hospital of Patra | 49 | 03/03 – 30/04 | It is a modern tertiary hospital, with about 800 beds and >30 specialized clinics, and serves > 1,500 patients a day. During the COVID-19 epidemic from February 2020, it became a reference center for Southern and Western Greece, serving a multitude of both externally confirmed cases and cases that required hospitalization in common wards as well as in ICUs. |
| Attikon GH | 40 | 03/01 – 05/15 | It is a 650-bed tertiary hospital in Western Attica. During the COVID-19 pandemic, the hospital was designated as a Covid-19 referral hospital. Confirmed cases were admitted in the Infectious Diseases Unit with a capacity of 8 isolated single-patient rooms or in dedicated hospital wards of 60 beds in total. Twenty ICU beds were also dedicated to Covid-19 in specific ICU areas with negative pressure. |
| General University Hospital of Larissa | 34 | 03/13 – 05/14 | The General University Hospital of Larissa is the referral center of the 5th Health Region of Central Greece for the management of COVID-19 patients, covering more than 1,000,000 population. Since March 2020, COVID-19 patients are managed in its Infectious Disease Unit. Patients were treated according to the therapeutic algorithms proposed by the Greek Committee of Public Health of the Ministry of Health, using hydroxychloroquine and azithomycin as the first-line main antiviral agents. |

**S1 Figure. Receiver operator curves (ROC) evaluating the model's performance on the testing set for patient subgroups.**

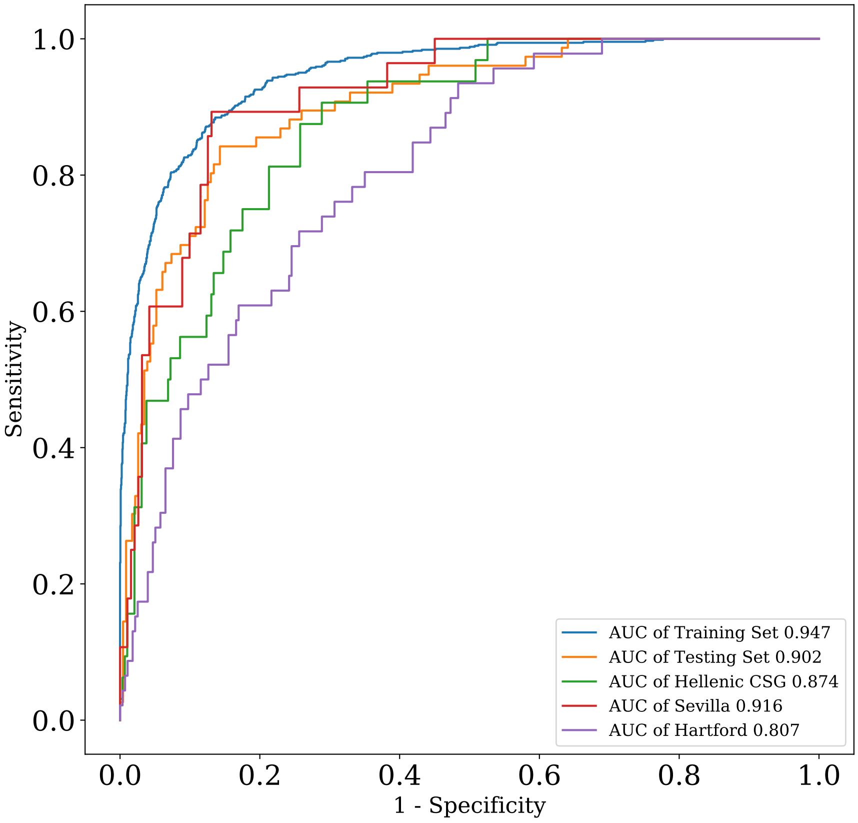


# References


[1] Troyanskaya O, Cantor M, Sherlock G, et al. Missing value estimation methods for DNA microarrays. Bioinformatics. 2001;17(6):520-525. doi:10.1093/bioinformatics/17.6.520

[2] Chen T, Guestrin C. XGBoost: A scalable tree boosting system. In: Proceedings of the ACM SIGKDD International Conference on Knowledge Discovery and Data Mining. Vol 13-17-Augu.; 2016:785-794. doi:10.1145/2939672.2939785

[3] Pedregosa F, Varoquaux G, Gramfort A, et al. Scikit-learn: Machine Learning in Python. J Mach Learn Res. 2011;12(85):2825-2830. http://jmlr.org/papers/v12/pedregosa11a.html.

[4] Akiba T, Sano S, Yanase T, Ohta T, Koyama M. Optuna: A Next-generation Hyperparameter Optimization Framework. In: Proceedings of the ACM SIGKDD International Conference on Knowledge Discovery and Data Mining. 2019:2623-2631. doi:10.1145/3292500.3330701

[5] Lundberg SM, Lee SI. A unified approach to interpreting model predictions. In: Advances in Neural Information Processing Systems. Vol 2017-Decem.; 2017:4766-4775. 14.

[6] Lundberg SM, Erion G, Chen H, et al. From local explanations to global understanding with explainable AI for trees. Nat Mach Intell. 2020;2(1):56-67. doi:10.1038/s42256-019-0138-9
