# Supplementary material for: COVID-19 mortality risk assessment: An international multi-center study
Source: PLoS One. 2020 Dec 9;15(12):e0243262. doi: 10.1371/journal.pone.0243262 (PMC7725386; doi:10.1371/journal.pone.0243262)
Supplement: S2 File — (PDF) [file pone.0243262.s002.pdf]

From predictions to prescriptions: A data-driven response to COVID-19

Dimitris Bertsimas[a,b,1], **Leonard Boussioux**[b], **Ryan Cory-Wright**[b], **Arthur Delarue**[b], **Vassilis Digalakis**[b], **Alexandre Jacquillat**[a,b], **Driss Lahlou Kitane**[b], **Galit Lukin**[b], **Michael Li**[b], **Luca Mingardi**[b], **Omid Nohadani**[c], **Agni Orfanoudaki**[b], **Theodore Papalexopoulos**[b], **Ivan Paskov**[b], **Jean Pauphilet**[b], **Omar Skali Lami**[b], **Bartolomeo Stellato**[b], **Hamza Tazi Bouardi**[b], **Kimberly Villalobos Carballo**[b], **Holly Wiberg**[b], **and Cynthia Zeng**[b]

[a]Sloan School of Management, Massachusetts Institute of Technology, Cambridge, MA 02142; [b]Operations Research Center, Massachusetts Institute of Technology, Cambridge, MA 02139; [c]Benefits Science Technologies, Boston, MA 02110

This manuscript was compiled on June 2, 2020

The COVID-19 pandemic has created unprecedented challenges worldwide. Strained healthcare providers make difficult decisions on patient triage, treatment and care management on a daily basis. Policy makers have imposed social distancing measures to slow the disease, at a steep economic price. We design analytical tools to support these decisions and combat the pandemic. Specifically, we propose a comprehensive data-driven approach to understand the clinical characteristics of COVID-19, predict its mortality, forecast its evolution, and ultimately alleviate its impact. By leveraging cohort-level clinical data, patient-level hospital data, and census-level epidemiological data, we develop an integrated four-step approach, combining descriptive, predictive and prescriptive analytics. First, we aggregate hundreds of clinical studies into the most comprehensive database on COVID-19 to paint a new macroscopic picture of the disease. Second, we build personalized calculators to predict the risk of infection and mortality as a function of demographics, symptoms, comorbidities, and lab values. Third, we develop a novel epidemiological model to project the pandemic's spread and inform social distancing policies. Fourth, we propose an optimization model to re-allocate ventilators and alleviate shortages. Our results have been used at the clinical level by several hospitals to triage patients, guide care management, plan ICU capacity, and re-distribute ventilators. At the policy level, they are currently supporting safe back-to-work policies at a major institution and equitable vaccine distribution planning at a major pharmaceutical company, and have been integrated into the US Center for Disease Control's pandemic forecast.

COVID-19 | Epidemiological modeling | Machine learning | Optimization

DRAFT

In just a few weeks, the whole world has been upended by the outbreak of COVID-19, an acute respiratory disease caused by a new coronavirus called SARS-CoV-2. The virus is highly contagious: it is easily transmitted from person to person via respiratory droplet nuclei and can persist on surfaces for days (1, 2). As a result, COVID-19 has spread rapidly—classified by the World Health Organization as a public health emergency on January 30, 2020 and as a pandemic on March 11. As of mid-May, over 4.5 million cases and 300,000 deaths have been reported globally (3).

Because no treatment is currently available, healthcare providers and policy makers are wrestling with unprecedented challenges. Hospitals and other care facilities are facing shortages of beds, ventilators and personal protective equipment—raising hard questions on how to treat COVID-19 patients with scarce supplies and how to allocate resources to prevent further shortages. At the policy level, most countries have imposed "social distancing" measures to slow the spread of the pandemic. These measures allow strained healthcare systems to cope with the disease by "flattening the curve" (4) but also come at a steep economic price (5, 6). Nearly all governments are now confronted to difficult decisions balancing public health and socio-economic outcomes.

This paper proposes a comprehensive data-driven approach to understand the clinical characteristics of COVID-19, predict its mortality, forecast its evolution, and ultimately alleviate its impact. We leverage a broad range of data sources, which include (i) our own cohort-level data aggregating hundreds of clinical studies, (ii) patient-level data obtained from electronic health records, and (iii) census reports on the scale of the pandemic. We develop an integrated approach spanning descriptive analytics (to derive a macroscopic understanding of the disease), predictive analytics (to forecast the near-term impact and longer-term dynamics of the pandemic), and prescriptive analytics (to support healthcare and policy decision-making).

Specifically, our approach comprises four steps (Figure 1):

- *Aggregating and visualizing the most comprehensive clinical database on COVID-19 (Section 1).* We aggregate cohort-level data on demographics, comorbidities, symptoms and lab values from 160 clinical studies. These data paint a broad picture of the disease, identifying common symptoms, disparities between mild and severe patients,

Significance Statement

In the midst of the COVID-19 pandemic, healthcare providers and policy makers are wrestling with unprecedented challenges. How to treat COVID-19 patients with equipment shortages? How to allocate resources to combat the disease? How to plan for the next stages of the pandemic? We present a data-driven approach to tackle these challenges. We gather comprehensive data from various sources, including clinical studies, electronic medical records, and census reports. We develop algorithms to understand the disease, predict its mortality, forecast its spread, inform social distancing policies, and re-distribute critical equipment. These algorithms provide decision support tools that have been deployed on our publicly available website, and are actively used by hospitals, companies, and policy makers around the globe.

D.B., R.C.W., A.D., A.J., D.L.K., M.L., O.N., A.O., I.P., J.P., O.S.L., B.S., H.T.B. and H.W. designed research; L.B., R.C.W., A.D., V.D., A.J., D.L.K., G.L., M.L., L.M., A.O., T.P., I.P., J.P., O.S.L., B.S., H.T.B., K.V.C., H.W. and C.Z. performed research; R.C.W., A.D., D.L.K., M.L., L.M., A.O., T.P., I.P., J.P., O.S.L., B.S., H.T.B., and H.W. analyzed data; D.B., R.C.W., A.D., A.J., M.L., O.N., A.O., J.P., H.T.B. and H.W. wrote the paper.

No author has any competing interest to declare.

[1]To whom correspondence should be addressed. E-mail: dbertsim@mit.edu

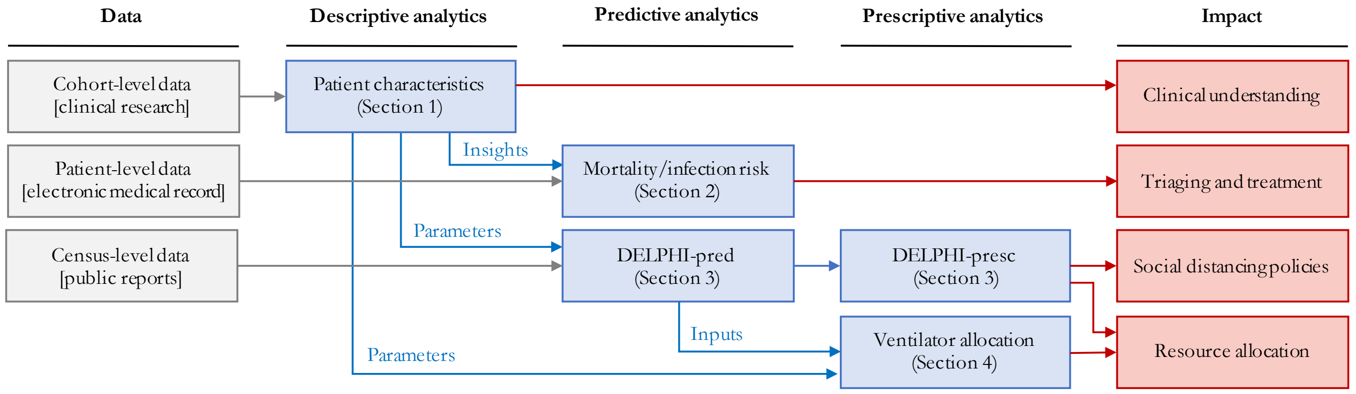

Fig. 1. Overview of our end-to-end analytics approach. We leverage diverse data sources to inform a family of descriptive, predictive and prescriptive tools for clinical and policy decision-making support.

and geographic disparities—insights that are hard to derive from any single study and can orient future clinical research on COVID-19, its mutations, and its disparate effects across ethnic groups.

- *Providing personalized indicators to assess the risk of mortality and infection (Section 2).* Using patient-level data, we develop machine learning models to predict mortality and infection risk, as a function of demographics, symptoms, comorbidities, and lab values. Using gradient boosting methods, the models achieve strong predictive performance—with an out-of-sample area under the curve above 90%. These models yield personalized calculators that can (i) guide triage, treatment, and care management decisions for strained healthcare systems, and (ii) serve as pre-screening tools for patients before they visit healthcare or testing facilities.
- *Developing a novel epidemiological model to forecast the evolution of the disease and assess the effects of social distancing (Section 3).* We propose a new compartmental model called DELPHI, which accounts for COVID-19 features such as underdetection and government response. The model estimates the disease's spread with high accuracy; notably, its projections from as early as April 3 have matched the number of cases observed in the United States up to mid-May. We also provide a data-driven assessment of social distancing policies, showing that the pandemic's spread is highly sensitive to the stringency and timing of mitigating measures.
- *Proposing an optimization model to support ventilator allocation in response to the pandemic (Section 4).* We formulate a mixed-integer optimization model to allocate ventilators efficiently in a semi-collaborative setting where resources can be shared both between healthcare facilities or through a central authority. In the United States, this allows us to study the trade-offs of managing the federal ventilator stockpile in conjunction with inter-state transfers. Results show that limited ventilator transfers could have eliminated shortages in April 2020.

A major contribution of our work is to treat these different questions as interdependent challenges raised by the pandemic—as opposed to a series of isolated problems. Indeed, clinical decision-making depends directly on patient inflows and available supplies, while resource planning and government responses react to patient-level outcomes. By combining various data sources into descriptive, predictive and prescriptive methods, this paper proposes an end-to-end approach to design a comprehensive and cohesive response to COVID-19.

Ultimately, this paper develops analytical tools to inform clinical and policy responses to the COVID-19 pandemic. These tools are available to the public on a dedicated website.* They have also been deployed in practice to combat the spread of COVID-19 globally. Several hospitals in Europe have used our risk calculators to support pre-triage and post-triage decisions, and a major financial institution in South America is applying our infection risk calculator to determine how employees can safely return to work. A major hospital system in the United States planned its intensive care unit (ICU) capacity based on our forecasts, and leveraged our optimization results to allocate ventilators across hospitals when the number of cases was rising. Our epidemiological predictions are used by a major pharmaceutical company to design a vaccine distribution strategy that can contain future phases of the pandemic. They have also been incorporated into the US Center for Disease Control's forecasts (7).

1. Descriptive Analytics: Clinical Outcomes Database

Early responses to the COVID-19 pandemic have been inhibited by the lack of available data on patient outcomes. Individual centers released reports summarizing patient characteristics. Yet, this decentralized effort makes it difficult to construct a cohesive picture of the pandemic.

To address this problem, we construct a database that aggregates demographics, comorbidities, symptoms, laboratory blood test results ("lab values", henceforth) and clinical outcomes from 160 clinical studies released between December 2019 and May 2020—made available on our website for broader use. The database contains information on 133,600 COVID-19 patients (3.13% of the global COVID-19 patients as of May 12, 2020), spanning mainly Europe (81,207 patients), Asia (19,418 patients) and North America (23,279 patients). To our knowledge, this is the largest dataset on COVID-19.

A. Data Aggregation. Each study was read by an MIT researcher, who transcribed numerical data from the manuscript. The appendix reports the main transcription assumptions.

*www.covidanalytics.io

DRAFT

Each row in the database corresponds to a cohort of patients—some papers study a single cohort, whereas others study several cohorts or sub-cohorts. Each column reports cohort-level statistics on demographics (e.g., average age, gender breakdown), comorbidities (e.g., prevalence of diabetes, hypertension), symptoms (e.g., prevalence of fever, cough), treatments (e.g., prevalence of antibiotics, intubation), lab values (e.g., average lymphocyte count), and clinical outcomes (e.g., average hospital length of stay, mortality rate). We also track whether the cohort comprises "mild" or "severe" patients (mild and severe cohorts are only a subset of the data).

Due to the pandemic's urgency, many papers were published before all patients in a cohort were discharged or deceased. Accordingly, we estimate the mortality rate from discharged and deceased patients only (referred to as "Projected Mortality").

B. Objectives. Our main goal is to leverage this database to derive a macroscopic understanding of the disease. We break it down into the following questions:

- Which symptoms are most prevalent?
- How do "mild" and "severe" patients differ in terms of symptoms, comorbidities, and lab values?
- Can we identify epidemiological differences in different parts of the world?

C. Descriptive Statistics. Table 1 depicts the prevalence of COVID-19 symptoms, in aggregate, classified into "mild" or "severe" patients, and classified per geographic region. Our key observations are that:

- Cough, fever, shortness of breath, and fatigue are the most prevalent symptoms of COVID-19.
- COVID-19 symptoms are much more diverse than those listed by public health agencies. COVID-19 patients can experience at least 15 different symptoms. In contrast, the US Center for Disease Control and Prevention lists seven symptoms (cough, shortness of breath, fever, chills, myalgia, sore throat, and loss of taste/smell) (8); the World Health Organization lists three symptoms (fever, cough, and fatigue) (9); and the UK National Health Service lists two main symptoms (fever and cough) (10). This suggests a lack of consensus among the medical community, and opportunities to revisit public health guidelines to capture the breadth of observed symptoms.
- Shortness of breath and elevated respiratory rates are much more prevalent in cases diagnosed as severe.
- Symptoms are quite different in Asia vs. Europe or North America. In particular, more than 75% of Asian patients experience fever, as compared to less than half in Europe and North America. Conversely, shortness of breath is much more prevalent in Europe and North America.

Using a similar nomenclature, Figure 2A reports demographics, comorbidities, lab values, and clinical outcomes (an extended version is available in the appendix). In terms of demographics, severe populations of patients have a higher incidence of male subjects and are older on average. Severe patients also have elevated comorbidity rates. Figures 2B and 2C visually confirm the impact of age and hypertension rates on population-level mortality—consistent with (11–13). In terms of lab values, CRP, AST, BUN, IL-6 and Protocalcitonin are highly elevated among severe patients.

D. Discussion and Impact. Our database is the largest available source of clinical information on COVID-19 assembled to date. As such, it provides new insights on common symptoms and the drivers of the disease's severity. Ultimately, this database can support guidelines from health organizations, and contribute to ongoing clinical research on the disease.

Another benefit of this database is its geographical reach. Results highlight disparities in patients' symptoms across regions. These disparities may stem from (i) different reporting criteria; (ii) different treatments; (iii) disparate impacts across different ethnic groups; and (iv) mutations of the virus since it first appeared in China. This information contributes to early evidence on COVID-19 mutations (14, 15) and on its disparate effects on different ethnic groups (16, 17).

Finally, the database provides average values of key parameters into our epidemiological model of the disease's spread and our optimization model of resource allocation (e.g., average length of stay of hospitalizations, average fraction of hospitalized patients put on a ventilator).

The insights derived from this descriptive analysis highlight the need for personalized data-driven clinical indicators. Yet, our population-level database cannot be leveraged directly to support decision-making at the patient level. We have therefore initiated a multi-institution collaboration to collect electronic medical records from COVID-19 patients and develop clinical risk calculators. These calculators, presented in the next section, are informed by several of our descriptive insights. Notably, the disparities between severe patients and the rest of the patient population inform the choice of the features included in our mortality risk calculator. Moreover, the geographic disparities suggest that data from Asia may be less predictive when building infection or mortality risk calculators designed for patients in Europe or North America—motivating our use of data from Europe.

2. Predictive Analytics: Mortality and Infection Risk

Throughout the COVID-19 crisis, physicians have made difficult triage and care management decisions on a daily basis. Oftentimes, these decisions could only rely on small-scale clinical tests, each requiring significant time, personnel and equipment and thus cannot be easily replicated. As the burden on "hot spots" has ebbed, hospitals began to aggregate rich data on COVID-19 patients. This data offers opportunities to develop algorithmic risk calculators for large-scale decision support—ultimately facilitating a more proactive and data-driven strategy to combat the disease globally.

We have established a patient-level database of thousands of COVID-19 hospital admissions. Using state-of-the-art machine learning methods, we develop a *mortality risk calculator* and an *infection risk calculator*. Together, these two risk assessments provide screening tools to support critical care management decisions, spanning patient triage, hospital admissions, bed assignment and testing prioritization. A more detailed model for mortality with lab values is presented in (18).

A. Methods. This investigation constitutes a multi-center study from healthcare institutions in Spain and Italy, two countries severely impacted by COVID-19. Specifically, we collected data from (i) Azienda Socio-Sanitaria Territoriale di Cremona (ASST Cremona), the main hospital network in the Province of Cremona, and (ii) HM Hospitals, a leading

DRAFT

Table 1. Count and prevalence of symptoms among COVID-19 patients, in aggregate, broken down into mild/severe patients, and broken down per continent (Asia, Europe, North America). Mild and severe patients only form a subset of the data, and so do patients from Asia, Europe and North America. A "-" indicates that fewer than 100 patients in a subpopulation reported on this symptom.

Symptom	All patients		Mild		Severe		Asia		Europe		North America	
	Count	(%)	Count	(%)	Count	(%)	Count	(%)	Count	(%)	Count	(%)
Cough	94,950	52.8%	6,833	63.0%	5,803	50.4%	14,034	56.2%	78,430	52.2%	1,113	63.6%
Fever	95,870	48.1%	6,864	79.3%	6,077	76.7%	14,750	76.6%	78,450	43.5%	1,481	41.3%
Short Breath	17,290	33.7%	6,006	16.1%	5,373	60.7%	11,330	19.7%	3,512	69.9%	1,111	49.2%
Fatigue	11,560	31.4%	5,313	35.3%	1,989	40.6%	11,320	30.8%	226	64.2%	–	–
Sputum	7,613	26.3%	4,995	29.2%	1,216	34.2%	7,395	26.7%	–	–	176	10.9%
Sore Throat	83,170	22.2%	3,513	14.2%	921	8.2%	6,013	10.4%	75,235	22.9%	550	9.8%
Myalgia	12,150	17.5%	4,455	16.4%	1,643	19.1%	8,517	15.5%	1,633	33.5%	755	25.3%
Elev. Resp. Rate	7,376	16.4%	527	9.7%	642	38.4%	1,257	14.6%	–	–	6,117	16.8%
Anorexia	3,928	15.8%	1,641	14.2%	808	15.4%	3,566	13.8%	312	40.5%	–	–
Headache	11,430	15.7%	5,068	12.2%	1,541	8.6%	7,929	9.9%	1,633	27.2%	551	8.7%
Nausea	10,070	12.4%	4,238	6.5%	1,798	5.6%	8,262	8.2%	312	22.4%	259	9.0%
Chest Pain	3,303	11.3%	767	12.2%	588	19.6%	2,984	12.2%	–	–	–	–
Diarrhea	16,520	11.1%	5,687	9.7%	5,369	9.0%	11,470	10.8%	3,512	10.4%	1,066	15.4%
Cong. Airway	1,639	8.7%	2,176	6.5%	234	14.1%	1,369	8.9%	–	–	258	7.4%
Chills	3,116	8.7%	2,751	9.9%	520	9.4%	2,794	8.2%	–	–	268	11.5%
Proj. Mortality	111,700	11.7%	7,428	0.4%	9,146	74.0%	12,820	16.7%	79,750	9.9%	19,060	15.8%

DRAFT

Feature	All	Mild	Severe
**Demographics**			
Male (%)	53.0%	48.8%	68.7%
Age (years)	51.3	46.1	68.2
White/European (%)	22.2%	9.7%	63.9%
African American (%)	5.4%	3.5%	2.5%
Asian (%)	51.3%	80.2%	31.2%
Hispanic/Latino	19.9%	0%	0%
Multiple ethnicities/other	3.6%	6.9%	2.7%
**Comorbidities**			
Smoking history	16.1%	12.2%	16.6%
Hypertension	35.9%	15.2%	54.4%
Diabetes	20.8%	6.8%	26.1%
Cardio Disease	12.4%	3.0%	20.3%
COPD	6.0%	2.8%	10.0%
Cancer	7.2%	3.2%	12.9%
Liver Disease	2.8%	2.3%	3.5%
Cebrovascular	9.8%	2.7%	24.8%
Kidney Disease	5.7%	1.2%	10.8%
**Lab values**			
White Blood Cells Count (WBC) (10^9/L)	6.41	5.07	6.80
Neutrophil Count (10^9/L)	4.72	5.12	5.78
Platelet Count (10^9/L)	195.7	184.0	170.4
Alanine Aminotransferase (ALT) (U/L)	29.0	24.6	31.1
Aspartate Aminotransferase (AST) (U/L)	37.3	27.1	45.7
Blood Urea Nitrogen Count (BUN) (mmol/L)	5.22	4.18	6.86
Creatinine (μmol/L)	63.08	66.0	56.4
C-Reactive Protein Count (CRP) (mg/L)	76.5	18.9	94.1
Interleukin-6 (IL-6) (pg/mL)	24.57	4.17	38.63
Procalcitonin (ng/mL)	2.26	1.85	4.81
Length of Stay (days)	10.7	14.0	7.97

A

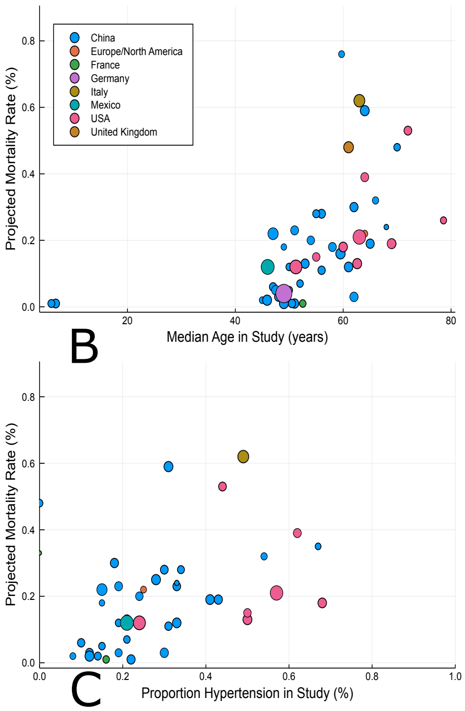

Fig. 2. Summary of demographics, comorbidities and lab values in mild and severe COVID-19 patients. (A) Comorbidities, demographics, average lab values, average length of stay and projected mortality among COVID-19 patients, in aggregate and broken down into mild/severe patients. (B) Impact of median age on projected mortality at a cohort level. (C) Impact of hypertension rates on projected mortality at a cohort level. The size of each dot represents the number of patients in the cohort, and its color represents the nation the study was performed in. We only include studies reporting both discharged and deceased patients.

hospital group in Spain with 15 general hospitals and 21 clinical centers spanning the regions of Madrid, Galicia, and León. We applied the following inclusion criteria to the calculators:

- **Mortality Risk**: We include adult patients diagnosed with COVID-19 and hospitalized. We consider patients who were either discharged from the hospital or deceased within the visit—excluding active patients. We include only lab values and vital values collected on the first day in the emergency department to match the clinical decision setting—predicting prognosis at the time of admission.

- **Infection Risk**: We include adult patients who underwent a polymerase chain reaction test for detecting

COVID-19 infection at the ASST Cremona hospital (19).[†] We include all patients, regardless of their clinical outcome. Each patient was subject to a blood test. We omit comorbidities since they are derived from the discharge diagnoses, hence not available for all patients.

We train two models for each calculator: one with lab values and one without lab values. Missing values are imputed using k-nearest neighbors imputation (20). We exclude features missing for more than 40% of patients. We train binary classification models for both risk calculators, using the XGBoost algorithm (21). We restrict the model to select at most 20 features, in order to make the resulting tool easily usable. We use SHapley Additive exPlanations (SHAP) (22, 23) to generate importance plots that identify risk drivers and provide transparency on the model predictions.

To evaluate predictive performance, we use 40 random data partitions into training and test sets. We compute the average Area Under the Curve (AUC), sensitivity, specificity, precision, negative predictive value, and positive predictive value. We calculate 95% confidence intervals using bootstrapping.

B. Results.

Study Population. The mortality study population comprises 2,831 patients, 711 (25.1%) of whom died during hospitalization while the remaining ones were discharged. The infection study population comprises 3,135 patients, 1,661 (53.0%) of whom tested positive for COVID-19. The full distributions of patient characteristics are reported in the appendix.

Performance Evaluation. All models achieve strong out-of-sample performance. Our mortality risk calculator has an AUC of 93.8% with lab values and 90.5% without lab values. Our infection risk calculator has an AUC of 91.8% with lab values and 83.1% without lab values. These values suggest a strong discriminative ability of the proposed models. We report in the appendix average results across all random data partitions.

We also report in the appendix threshold-based metrics, which evaluate the discriminative ability of the calculators at a fixed cutoff. With the threshold set to ensure a sensitivity of at least 90% (motivated by the high costs of false negatives), we obtain accuracies spanning 65%–80%.

The mortality model achieves better overall predictive performance than the infection model. As expected, both models have better predictive performance with lab values than without lab values. Yet, the models without lab values still achieve strong predictive performance.

Model Interpretation. Figure 3 plots the SHAP importance plots for all models. The figures sort the features by decreasing significance. For each one, the row represents its impact on the SHAP value, as the feature ranges from low (blue) to high (red). Higher SHAP values correspond to increased likelihood of a positive outcome (i.e. mortality or infection). Features with the color scale oriented blue to red (resp. red to blue) from left to right have increasing (resp. decreasing) risk as the feature increases. For example, "Age" is the most important feature of the mortality score with lab values (Figure 3A), and older patients have higher predicted mortality.

[†] HM Hospitals patients were not included since no negative case data was available.

C. Discussion and Impact.

The models with lab values provide algorithmic screening tools that can deliver COVID-19 risk predictions using common clinical features. In a constrained healthcare system or in a clinic without access to advanced diagnostics, clinicians can use these models to rapidly identify high-risk patients to support triage and treatment decisions.

The models without lab values offer an even simpler tool that could be used outside of a clinical setting. In strained healthcare systems, it can be difficult for patients to obtain direct advice from providers. Our tool could serve as a pre-screening step to identify personalized infection risk—without visiting a testing facility. While the exclusion of lab values reduces the AUC (especially for infection), these calculators still perform strongly.

Our models provide insights into risk factors and biomarkers related to COVID-19 infection and mortality. Our results suggest that the main indicators of mortality risk are age, BUN, CRP, AST, and low oxygen saturation. These findings validate several population-level insights from Section 1 and are in agreement with clinical studies: prevalence of shortness of breath (24), elevated levels of CRP as an inflammatory marker (25, 26), and elevated AST levels due to liver dysfunction in severe COVID-19 cases (11, 27).

Turning to infection risk, the main indicators are CRP, WBC, Calcium, AST, and temperature. These findings are also in agreement with clinical reports: an elevated CRP generally indicates an early sign of infection and implies lung lesions from COVID-19 (28), elevated levels of leukocytes suggest cytokine release syndrome caused by SARS-CoV-2 virus (29), and lowered levels of serum calcium signal higher rate of organ injury and septic shock (30). The agreement between our findings and clinicl observations offers credibility for the use of our calculators to support clinical decision-making—although they are not intended to substitute clinical diagnostic or medical expertise.

When lab values are not available, the widely accepted risk factors of age, oxygen saturation, temperature, and heart rate become the key indicators for both risk calculators. We observe that mortality risk is higher for male patients (blue in Figure 3B) than for female patients (red), confirming clinical reports (31, 32). An elevated respiratory frequency becomes an important predictor of infection, as reported in (33). These findings suggest that demographics and vitals provide valuable information in the absence of lab values. However, when lab values are available, these other features become secondary.

A limitation of the current mortality model is that it does not take into account medication and treatments during hospitalization. We intend to incorporate these in future research to make these models more actionable. Furthermore, these models aim to reveal associations between risks and patient characteristics but are not designed to establish causality.

Overall, we have developed data-driven calculators that allow physicians and patients to assess mortality and infection risks in order to guide care management—especially with scarce healthcare resources. These calculators are being used by several hospitals within the ASST Cremona system to support triage and treatment decisions—alleviating the toll of the pandemic. Our infection calculator also supports safety protocols for Banco de Credito del Peru, the largest bank in Peru, to determine how employees can return to work.

DRAFT

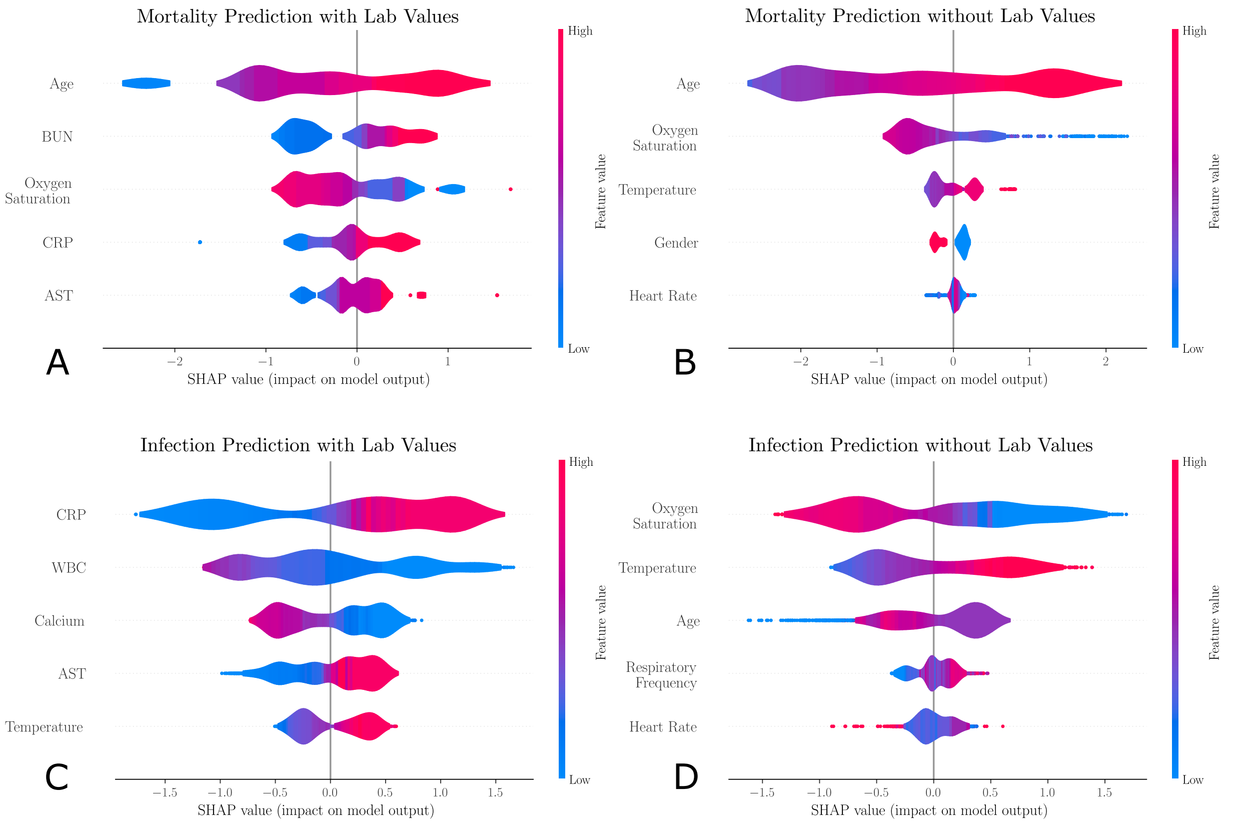

Fig. 3. SHapley Additive exPlanations (SHAP) importance plots for the mortality and infection risk calculators, including: (A) the mortality model with lab values; (B) the mortality model without lab values; (C) the infection model with lab values; and (D) the infection model without lab values. The five most important features are shown for each model. Gender is a binary feature (female is equal to 1, shown in red; male is equal to 0, shown in blue). Each row represents the impact of a feature on the outcome, with higher SHAP values indicating higher likelihood of a positive outcome.

3. Predictive and Prescriptive Analytics: Disease Projections and Government Response

We develop a new epidemiological model, called DELPHI (Differential Equations Leads to Predictions of Hospitalizations and Infections). The model first provides a predictive tool to forecast the number of detected cases, hospitalizations and deaths—we refer to this model as "DELPHI-pred". It then provides a prescriptive tool to simulate the effect of policy interventions and guide government response to the COVID-19 pandemic—we refer to this model as "DELPHI-presc". All models are fit in each US state (plus the District of Columbia). A detailed presentation and discussion on the implications of the DELPHI model especially with respect to government interventions is presented in (34).

A. DELPHI-pred: Projecting Early Spread of COVID-19.

A.1. Model Development. DELPHI is a compartmental model, with dynamics governed by ordinary differential equations. It extends the standard SEIR model by defining 11 states (Figure 4A): susceptible (S), exposed (E), infectious (I), undetected people who will recover (U_R) or decease (U_D), detected hospitalized people who will recover (DH_R) or decease (DH_D), quarantined people who will recover (DQ_R) or decease (DQ_D), recovered (R) and deceased (D). The separation of the U_R/U_D, DQ_R/DQ_D and DH_R/DH_D states enables separate fitting of recoveries and deaths from the data.

As opposed to other COVID-19 models (see, e.g., 35), DELPHI captures two key elements of the pandemic:

- **Underdetection**: Many cases remain undetected due to limited testing, record failures, and detection errors. Ignoring them would underestimate the scale of the pandemic. We capture them through the U_R and U_D states.
- **Government Response**: "Social distancing" policies limit the spread of the virus. Ignoring them would overestimate the spread of the pandemic. We model them through a decline in the infection rate over time. Specifically, we write: $\frac{\mathrm{d}S}{\mathrm{d}t} = -\alpha\gamma(t)S(t)I(t)$, where α is a constant baseline rate and $\gamma(t)$ is a time-dependent function characterizing each state's policies, modeled as follows:

$$\gamma(t) = \frac{2}{\pi}\arctan\left(\frac{-(t-t_0)}{k}\right) + 1.$$

The inverse tangent function provides a concave-convex relationship, capturing three phases of government response. In *Phase I*, most activities continue normally as people adjust their behavior. In *Phase II*, the infection rate declines sharply as policies are implemented. In *Phase III*, the decline in the infection rate reaches saturation. The parameters t_0 and k can be respectively thought of as the start date and the strength of the response.

Ultimately, DELPHI involves 13 parameters that define the transition rates between the 11 states. We calibrate six of them from our clinical outcomes database (Section 1). Using non-linear optimization, we estimate seven parameters for each US state from the data to minimize in-sample error. This training procedure leverages historical data on the number of cases and deaths per US county (36). We include each state as soon as it records more than 100 cases. We provide details on the fitting procedure in the appendix.

A.2. Validation. DELPHI was created in late March and has been continuously updated to reflect new observed data. Figure 4B shows our projections made on three different dates, and compares them against historical observations. This plot focuses on the number of cases, but a similar plot for the number of deaths is reported in the appendix.

In addition to providing aggregate validation figures, we also evaluate the model's out-of-sample performance quantitatively, using a backtesting procedure. To our knowledge, this represents the first attempt to assess the predictive performance of COVID-19 projections. Specifically, we fit the model's parameters using data up to April 27, build projections from April 28 to May 12, and evaluate the resulting Mean Absolute Percentage Error (MAPE). Figure 4C reports the results in each US state.

A.3. Discussion and Impact. Results suggest that DELPHI-pred achieves strong predictive performance. The model has been consistently predicting, with high accuracy the overall spread of the disease for several weeks. Notably, DELPHI-pred was able to anticipate, as early as April 3rd, the dynamics of the pandemic in the United States up to mid-May. At a time where 200,000–300,000 cases were reported, the model was predicting 1.2M–1.4M cases by mid-May—a prediction that proved accurate 40 days later.

Our quantitative results confirm the visual evidence. The MAPE is small across US states. The median MAPE is 8.5% for the number of cases—the 10% and 90% percentiles are equal to 1.9% and 16.7%. The median MAPE is 7.8% for the number of deaths—the 10% and 90% percentiles are equal to 3.3% and 25.1%. Given the high level of uncertainty and variability in the disease's spread, this level of accuracy is suggestive of excellent out-of-sample performance.

As Figure 4C shows, a limitation of our model is that the relative error remains large for a small minority of US states. These discrepancies stem from two main reasons. First, errors are typically larger for states that have recorded few cases (WY) or few deaths (AK, KS, NE). Like all SEIR-derived models, DELPHI performs better on large populations. Moreover, the MAPE metric emphasizes errors on smaller population counts. Second, our model is fitted at the state level, implicitly assuming that the spread of the pandemic is independent from one state to another—thus ignoring inter-state travel. This limitation helps explain the above-median error in a few heartland states which were confronted to the pandemic in later stages (MN, TN, IA).

In summary, DELPHI-pred is a novel epidemiological model of the pandemic, which provides high-quality estimates of the daily number of cases and deaths per US state. This model has been incorporated to the forecasts used by the US Center for Disease Control to chart and anticipate the spread of the pandemic (7). It has also been used by the Hartford HealthCare system—the major hospital system in Connecticut, US—to plan its ICU capacity, and by a major pharmaceutical company to design a vaccine distribution strategy that can most effectively contain the next phases of the pandemic.

B. DELPHI-presc: Toward Re-opening Society. To inform the relaxation of social distancing policies, we link policies to the infection rate using machine learning. Specifically, we predict the values of $\gamma(t)$, obtained from the fitting procedure of DELPHI-pred. For simplicity and interpretability, we consider a simple model based on regression trees (37) and restrict the independent variables to the policies in place. We classify policies based on whether they restrict mass gatherings, school and/or other activities (referred to as "Others", and including business closures, severe travel limitations and/or closing of non-essential services). We define a set of seven mutually exclusive and collectively exhaustive policies observed in the US data: (i) *No measure*; (ii) *Restrict mass gatherings*; (iii) *Restrict others*; (iv) *Authorize schools, restrict mass gatherings and others*; (v) *Restrict mass gatherings and schools*; (vi) *Restrict mass gatherings, schools and others*; and (vii) *Stay-at-home*.

We report the regression tree in the appendix, obtained from state-level data in the United States. This model achieves an out-of-sample R^2 of 0.8, suggesting a good fit to the data. As expected, more stringent policies lead to lower values of $\gamma(t)$. The results also provide comparisons between various policies—for instance, school closures seem to induce a stronger reduction in the infection rate than restricting "other" activities. More importantly, the model quantifies the impact of each policy on the infection rate. We then use these results to predict the value of $\gamma(t)$ as a function of the policies (see appendix for details), and simulate the spread of the disease as states progressively loosen social distancing policies.

Figure 4D plots the projected case count in the state of New York (NY), for different policies (we report a similar plot for the death count in the appendix). Note that the stringency of the policies has a significant impact on the pandemic's spread and ultimate toll. For instance, relaxing all social distancing policies on May 12 can increase the *cumulative* number of cases in NY by up to 25% by September.

Using a similar nomenclature, Figure 4E shows the case count if all social distancing policies are relaxed on May 12 vs. May 26. Note that the timing of the policies also has a strong impact: a two-week delay in re-opening society can greatly reduce a resurgence in NY.

The road back to a new normal is not straightforward: results suggest that the disease's spread is highly sensitive to both the intensity and the timing of social distancing policies. As governments grapple with an evolving pandemic, DELPHI-presc can be a useful tool to explore alternative scenarios and ensure that critical decisions are supported with data.

4. Prescriptive Analytics: Ventilator Allocation

COVID-19 is primarily an acute respiratory disease. The World Health Organization recommends that patients with oxygen saturation levels below 93% receive respiratory support (9). Following the standard Acute Respiratory Distress Syndrome protocol, COVID-19 patients are initially put in the prone position and then put in a drug induced paralysis via a neuromuscular blockade to prevent lung injury (38). Patients

DRAFT

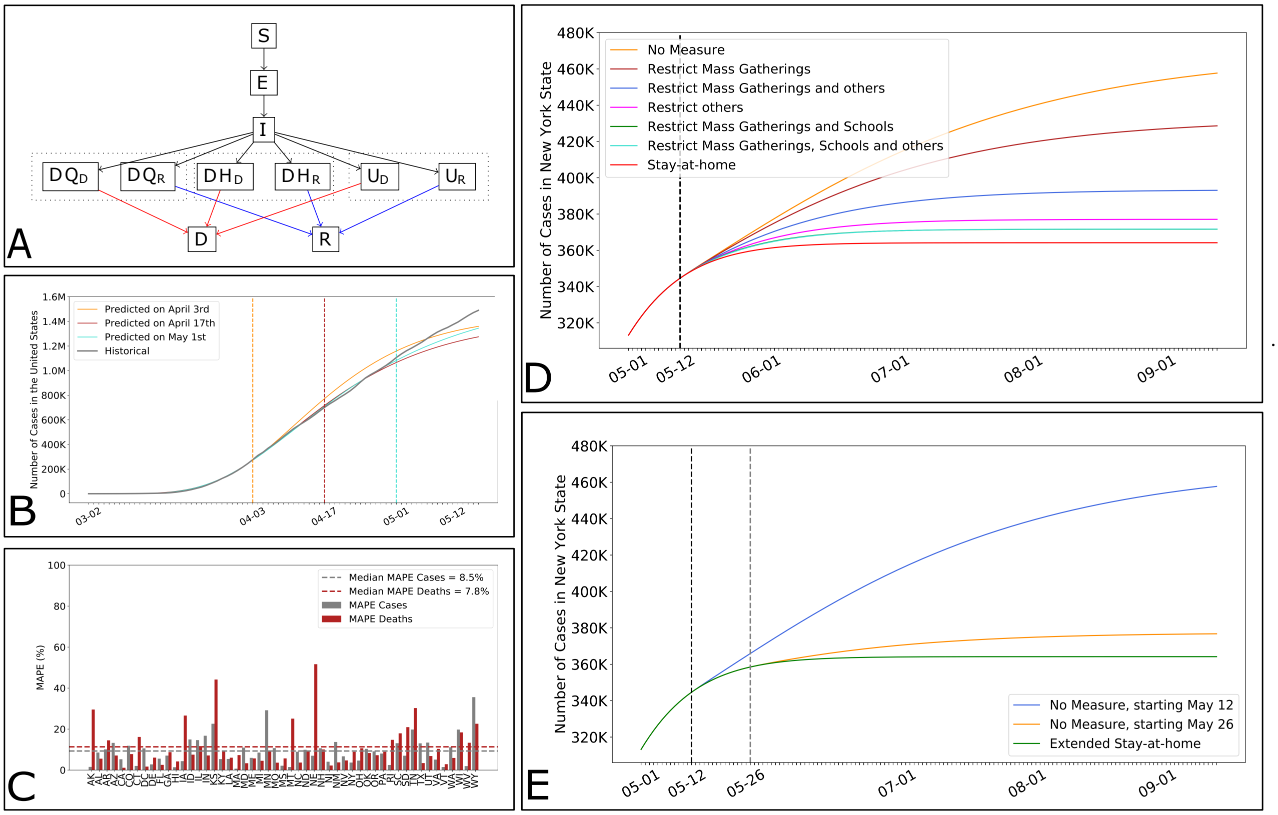

Fig. 4. DELPHI, an epidemiological model to guide government response. (A) Simplified flow diagram of DELPHI. (B) Cumulative number of cases in the United States according to our projections made at different points in time, against actual observations. (C) Out-of-sample Mean Absolute Percentage Error (MAPE) on the number of cases and deaths per US state. (D) Impact of different policies on the future number of cases, in NY. (E) Impact of the timing of policies on the future number of cases, in NY.

are then put on a ventilator, which delivers high concentrations of oxygen while removing carbon dioxide (39). Early evidence suggests that ventilator intubation reduces the risk of hypoxia for COVID-19 patients (40).

As a result, hospitals have been facing ventilator shortages worldwide (41). Still, local shortages do not necessarily imply global shortages. For instance, in April 2020, the total supply of ventilators in the United States exceeded the projected demand from COVID-19 patients. Ventilator shortages could thus be alleviated by pooling the supply, i.e., by strategically allocating the surge supply of ventilators from the federal government and facilitating inter-state transfers of ventilators.

We propose an optimization model to support the allocation of ventilators in a semi-collaborative setting where resources can be shared both between healthcare facilities or through a central authority. Based on its primary motivation, we formulate the model to support the management of the federal supply of ventilators and inter-state ventilator transfers in the United States. A similar model has also been used to support inter-hospital transfers of ventilators. The model can also support inter-country ventilator allocation during the next phases of the pandemic. This model leverages the demand projections from DELPHI-pred (Section 3) to prescribe resource allocation recommendations—with the ultimate goal of alleviating the health impact of the pandemic.

A. Model. Resource allocation is critical when clinical care depends on scarce equipment. Several studies have used optimization to support ventilator pooling. A time-independent model was first developed for influenza planning (42). A time-dependent stochastic optimization model was developed to support transfers to and from the federal government for COVID-19, given scenarios regarding the pandemic's spread (43). In this section, we propose a deterministic time-dependent model, leveraging the projections from DELPHI-pred.

We model ventilator pooling as a multi-period resource allocation over S states and D days. The model takes as input ventilator demand in state s and day d, denoted as $v_{s,d}$, as well as parameters capturing the surge supply from the federal government and the extent of inter-state collaboration. We formulate an optimization problem that decides on the number of ventilators transferred from state s to state s' on day d, and on the number of ventilators allocated from the federal government to state s on day d. We propose a bi-objective formulation. The first objective is to minimize ventilator-day shortages; for robustness, we consider both projected shortages (based on demand forecasts) and worst-case shortages (including a buffer in the demand estimates). The second objective is to minimize inter-state transfers, to limit the operational and political costs of inter-state coordination. Mixed-integer optimization provides modeling flexibility to capture spatial-temporal dynamics and the trade-offs between these various objectives. We report the mathematical formulation of the model, along with the key assumptions, in the appendix.

B. Results. We implemented the model on April 15, a time of pressing ventilator need in the United States. We estimate the number of hospitalizations from DELPHI-pred as the sum

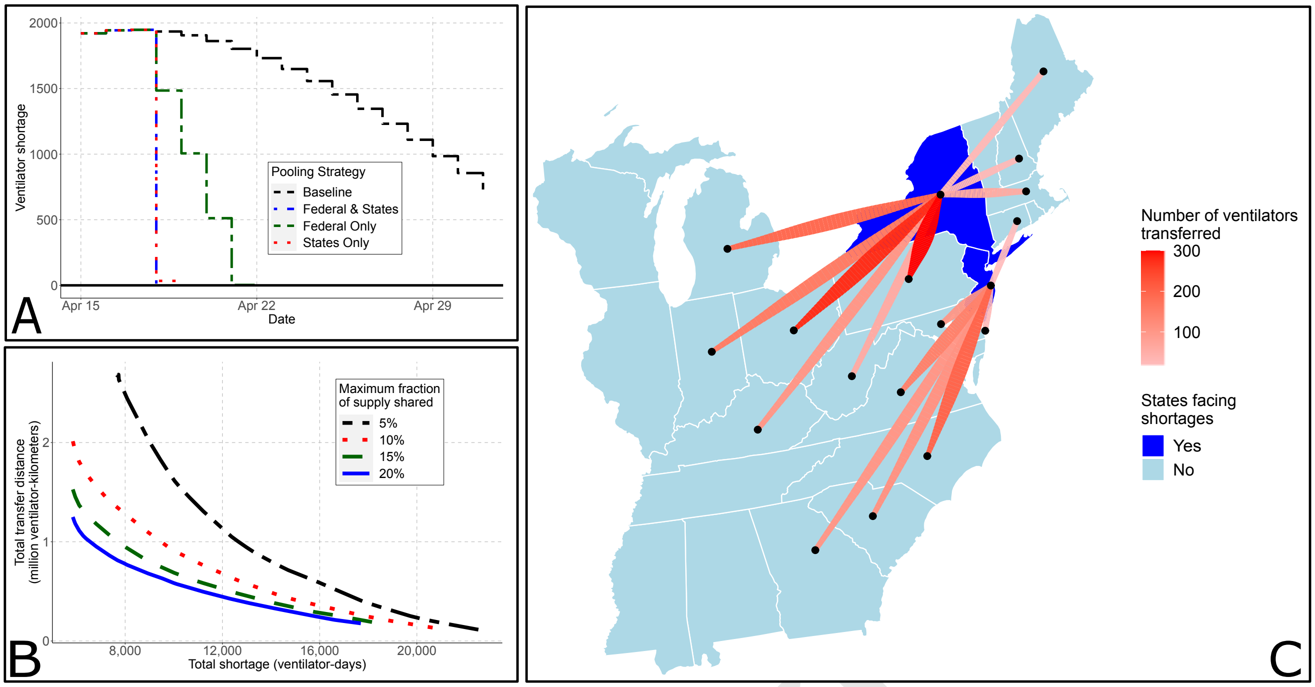

Fig. 5. The edge of optimization to eliminate ventilator shortages. (A) Projected shortages (in ventilator-days) in a baseline setting (without transfers) and with optimized transfers between the states and/or from the federal government. (B) Pareto frontier between transfer distance and total shortage, for different state pooling fractions. (C) Map of inter-state transfers recommended on April 15 in the US Northeast. For clarity, we do not plot shortages of fewer than 5 ventilators and transfers of fewer than 10.

DRAFT

of DH_R and DH_D. From our clinical outcomes database in Section 1, we estimate that 25% of hospitalized patients are put on a ventilator, which we use to estimate the demand for ventilators. We also obtain the average length of stay from our clinical outcomes database (Figure 2).

Figure 5A shows the evolution of ventilator shortages with and without ventilator transfers from the federal government and inter-state transfers. These results indicate that ventilator pooling can rapidly eliminate all ventilator shortages. Figure 5C shows ventilator transfers recommended in the US Northeast on April 15 (with inter-state transfers only), overlaid on a map displaying the predicted shortage without transfers.

There are different pathways toward eliminating ventilator shortages. Figure 5B shows the trade-off between shortages and transfer distance—each line corresponds to the maximal fraction of its own ventilators that each state can pool. Overall, states do not have to share more than 10% of their supply at any time to efficiently eliminate shortages. States can largely meet their needs with help from neighboring states, with cross-country transfers only used as a last resort. Broadly, results underscore trade-offs between ventilator shortages, the extent of inter-state transfers, the number of ventilators allocated from the federal government, and the robustness of the solution. We discuss these trade-offs further in the appendix.

C. Discussion and Impact. Our main insight is that ventilator shortages could be eliminated altogether through inter-state transfers and strategic management of the federal supply. Results also underscore (i) the benefits of inter-state coordination and (ii) the benefits of early coordination. First, ventilator shortages can be eliminated through inter-state transfers alone: leveraging a surge supply from the federal government is not required, though it may reduce inter-state transfers. Under our recommendation, the most pronounced transfers occur from states facing no shortages (Ohio, Pennsylvania, and North Carolina) to states facing strong shortages (New York, New Jersey). Second, most transfers occur in early stages of the pandemic. This underscores the benefits of leveraging a predictive model like DELPHI-pred to align the ventilator supply with demand projections as early as possible.

A similar model has been developed to support the redistribution of ventilators across hospitals within the Hartford HealthCare system in Connecticut—using county-level forecasts of ventilator demand obtained from DELPHI-pred. This model has been used by a collection of hospitals in the United States to align ventilator supply with projected demand at a time where the pandemic was on the rise.

Looking ahead, the proposed model can support the allocation of critical resources in the next phases of the pandemic—spanning ventilators, medicines, personal protective equipment etc. Since epidemics do not peak in each state at the same time, states whose infection peak has already passed or lies weeks ahead can help other states facing immediate shortages at little costs to their constituents. Inter-state transfers of ventilators occurred in isolated fashion through April 2020; our model proposes an automated decision-making tool to support these decisions systematically. As our results show, proactive coordination and resource pooling can significantly reduce shortages—thus increasing the number of patients that can be treated without resorting to extreme clinical recourse with side effects (such as splitting ventilators).

5. Conclusion

This paper proposes a comprehensive data-driven approach to address several core challenges faced by healthcare providers

and policy makers in the midst of the COVID-19 pandemic. We have gathered and aggregated data from hundreds of clinical studies, electronic health records, and census reports. We have developed descriptive, predictive and prescriptive models, combining methods from machine learning, epidemiology, and mixed-integer optimization. Results provide insights on the clinical aspects of the disease, on patients' infection and mortality risks, on the dynamics of the pandemic, and on the levers that policy makers and healthcare providers can use to alleviate its toll. The models developed in this paper also yield decision support tools that have been deployed on our dedicated website and that are actively being used by several hospitals, companies and policy makers.

Acknowledgments

We would like to thank Dr. Barry Stein, Dr. Ajay Kumar, Dr. Rocco Orlando, and Michelle Schneider from the Hartford HealthCare system, Dr. Angelo Pan, Dr. Rosario Canino, Sophie Testa and Federica Pezzetti from ASST Cremona, and HM Hospitals for discussions and data, as well as Hari Bandi, Katherine Bobroske, Martina Dal Bello, Mohammad Fazel-Zarandi, Alvaro Fernandez Galiana, Samuel Gilmour, Adam Kim, Zhen Lin, Liangyuan Na, Matthew Sobiesk, Yuchen Wang and Sophia Xing from our extended team for helpful discussions.

1. G Kampf, D Todt, S Pfaender, E Steinmann, Persistence of coronaviruses on inanimate surfaces and its inactivation with biocidal agents. *J. Hosp. Infect.* (2020).
2. S Sanche1, et al., High Contagiousness and Rapid Spread of Severe Acute Respiratory Syndrome Coronavirus 2. *Emerg. Infect. Dis.* **27** (2020).
3. John Hopkins University, Center for Systems Science and Engineering (https://systems.jhu.edu/research/public-health/ncov/) (2020).
4. RM Anderson, H Heesterbeek, D Klinkenberg, TD Hollingsworth, How will country-based mitigation measures influence the course of the covid-19 epidemic? *The Lancet* **395**, 931–934 (2020).
5. N Fernandes, Economic effects of coronavirus outbreak (covid-19) on the world economy. *Available at SSRN 3557504* (2020).
6. WJ McKibbin, R Fernando, The global macroeconomic impacts of covid-19: Seven scenarios. *CAMA Work. Pap.* (2020).
7. US Center for Disease Control, COVID-19 Forecasts (https://www.cdc.gov/coronavirus/2019-ncov/covid-data/forecasting-us.html) (2020).
8. US Center for Disease Control, Symptoms of Coronavirus (https://www.cdc.gov/coronavirus/2019-ncov/symptoms-testing/symptoms.html) (2020) (Accessed 11 May 2020).
9. World Health Organization, Coronavirus (https://www.who.int/health-topics/coronavirus) (2020) (Accessed 11 May 2020).
10. National Health Service, Check if you have Coronavirus symptoms (https://www.nhs.uk/conditions/coronavirus-covid-19/check-if-you-have-coronavirus-symptoms) (2020) (Accessed 11 May 2020).
11. Wj Guan, et al., Clinical characteristics of coronavirus disease 2019 in china. *New Engl. journal medicine* **382**, 1708–1720 (2020).
12. P Goyal, et al., Clinical characteristics of Covid-19 in New York City. *New Engl. J. Medicine* (2020).
13. CM Petrilli, et al., Factors associated with hospitalization and critical illness among 4,103 patients with covid-19 disease in new york city. *medRxiv* (2020).
14. P Forster, L Forster, C Renfrew, M Forster, Phylogenetic network analysis of SARS-CoV-2 genomes. *Proc. Natl. Acad. Sci.* **117**, 9241–9243 (2020).
15. LA Holland, et al., An 81 nucleotide deletion in SARS-CoV-2 ORF7a identified from sentinel surveillance in Arizona (Jan-Mar 2020). *J. Virol.* (2020).
16. S Garg, Hospitalization rates and characteristics of patients hospitalized with laboratory-confirmed coronavirus disease 2019—covid-net, 14 states, march 1–30, 2020. *MMWR. Morb. Mortal. Wkly. Rep.* **69** (2020).
17. FS Vahidy, et al., Racial and ethnic disparities in sars-cov-2 pandemic: Analysis of a covid-19 observational registry for a diverse us metropolitan population. *medRxiv* (2020).
18. D Bertsimas, et al., Covid-19 Mortality Risk Assessment: An international multi-center study. (2020) submitted for publication.
19. L Lan, et al., Positive RT-PCR test results in patients recovered from COVID-19. *Jama* **323**, 1502–1503 (2020).
20. O Troyanskaya, et al., Missing value estimation methods for dna microarrays. *Bioinformatics* **17**, 520–525 (2001).
21. T Chen, C Guestrin, Xgboost: A scalable tree boosting system in *Proceedings of the 22nd acm sigkdd international conference on knowledge discovery and data mining.* pp. 785–794 (2016).
22. SM Lundberg, SI Lee, A unified approach to interpreting model predictions in *Advances in Neural Information Processing Systems 30*, eds. I Guyon, et al. (Curran Associates, Inc.), pp. 4765–4774 (2017).
23. SM Lundberg, et al., From local explanations to global understanding with explainable AI for trees. *Nat. Mach. Intell.* **2**, 2522–5839 (2020).
24. Y Wang, Y Wang, Y Chen, Q Qin, Unique epidemiological and clinical features of the emerging 2019 novel coronavirus pneumonia (covid-19) implicate special control measures. *J. medical virology* **92**, 568–576 (2020).
25. TP Velavan, CG Meyer, The covid-19 epidemic. *Trop. medicine & international health* **25**, 278 (2020).
26. D Caruso, et al., Chest ct features of covid-19 in rome, italy. *Radiology*, 201237 (2020).
27. C Huang, et al., Clinical features of patients infected with 2019 novel coronavirus in Wuhan, China. *The Lancet* **395**, 497–506 (2020).
28. W Ling, C-reactive protein levels in the early stage of COVID-19. *Med. et Maladies Infect.* (2020).
29. Y Shi, et al., COVID-19 infection: the perspectives on immune responses (2020).
30. Q Shi, et al., Serum calcium as a biomarker of clinical severity and prognosis in patients with coronavirus disease 2019: a retrospective cross-sectional study. *Critical Care Emerg. Medicine* (2020).
31. RE Jordan, P Adab, K Cheng, Covid-19: risk factors for severe disease and death (2020).
32. Y Liu, et al., Neutrophil-to-lymphocyte ratio as an independent risk factor for mortality in hospitalized patients with covid-19. *J. Infect.* (2020).
33. F Zhou, et al., Clinical course and risk factors for mortality of adult inpatients with covid-19 in wuhan, china: a retrospective cohort study. *The Lancet* (2020).
34. ML Li, et al., Forecasting COVID-19 and Analyzing the Effect of Government Interventions. (2020) submitted for publication.
35. SM Kissler, C Tedijanto, E Goldstein, YH Grad, M Lipsitch, Projecting the transmission dynamics of sars-cov-2 through the postpandemic period. *Science* (2020).
36. New York Times, Coronavirus in the U.S.: Latest Map and Case Count (https://www.nytimes.com/interactive/2020/us/coronavirus-us-cases.html) (2020).
37. L Breiman, J Friedman, CJ Stone, RA Olshen, *Classification and regression trees.* (CRC press), (1984).
38. RA Cornejo, et al., Effects of prone positioning on lung protection in patients with acute respiratory distress syndrome. *Am. journal respiratory critical care medicine* **188**, 440–448 (2013).
39. T Bein, et al., The standard of care of patients with ARDS: ventilatory settings and rescue therapies for refractory hypoxemia. *Intensive care medicine* **42**, 699–711 (2016).
40. L Meng, et al., Intubation and Ventilation amid the COVID-19 Outbreak Wuhan's Experience. *Anesthesiol. The J. Am. Soc. Anesthesiol.* (2020).
41. ML Ranney, V Griffeth, AK Jha, Critical supply shortages—the need for ventilators and personal protective equipment during the Covid-19 pandemic. *New Engl. J. Medicine* (2020).
42. HC Huang, et al., Stockpiling ventilators for influenza pandemics. *Emerg. infectious diseases* **23**, 914 (2017).
43. S Mehrotra, H Rahimian, M Barah, F Luo, K Schantz, A model of supply-chain decisions for resource sharing with an application to ventilator allocation to combat COVID-19. *Nav. Res. Logist. (NRL)* (2020).

DRAFT

www.pnas.org

Supplementary Information for

From predictions to prescriptions: A data-driven response to COVID-19

Dimitris Bertsimas, Leonard Boussioux, Ryan Cory-Wright, Arthur Delarue, Vassilis Digalakis, Alexandre Jacquillat, Driss Lahlou Kitane, Galit Lukin, Michael Li, Luca Mingardi, Omid Nohadani, Agni Orfanoudaki, Theodore Papalexopoulos, Ivan Paskov, Jean Pauphilet, Omar Skali Lami, Bartolomeo Stellato, Hamza Tazi Bouardi, Kimberly Villalobos Carballo, Holly Wiberg and Cynthia Zeng

Dimitris Bertsimas.
E-mail: dbertsim@mit.edu

This PDF file includes:

Supplementary text
Figs. S1 to S7
Tables S1 to S6
SI References

Supporting Information Text

1. Supplementary Materials on Clinical Outcomes Database

The clinical outcomes database analyzed in Section 1 aggregates reported data from different hospitals across the world. These hospitals may have different equipment and reporting standards. It was obtained through a human reading process, which is inherently imperfect. For this reason, we now list some important observations and caveats about the database, and refer the reader to our website for a complete list of the 160 papers entered into the database.[*]

- To minimize human error in data reporting, we have verified some key features with additional scrutiny, including mortality, ICU and hospital length of stay, key symptoms (fever, cough, short breath, fatigue, diarrhea) and common comorbidities (hypertension, diabetes).

- Across papers, subcohort divisions may follow different criteria, including: severity of disease (severe vs. mild), mortality (survivors vs. non-survivors), treatment (intubation vs. non-intubation), comorbidity (diabetic vs. non-diabetic). To retain a large enough number of studies in each category, we classify a population as "mild" if the study classifies it as "not asymptomatic" and "mild", "general", or "non-ICU" and not "severe/critical"; and we classify a population as "severe" if the study classifies it as "severe", "critical", "ICU only" or "non-survivors only".

- Studies in this dataset do not always have the same purpose. For instance, many papers from Italy seem to report data only on non-survivors. In addition, some studies focus on the disease's contagion profile, with little information on mortality, discharge, stay length. Data points from these studies may exhibit a high proportion of missing features.

- We have tried to report all lab values in consistent units. We have included a companion document (Reference Ranges) with corresponding reference ranges to facilitate analysis. There are some instances where the reported lab units seem inconsistent with the expected ranges (e.g. for D-Dimer), but we have generally reported the raw values from the source papers.

- The papers entered in the database do not consistently report confidence intervals alongside population means. For this reason, we have declined to provide confidence intervals for the quantities estimated in this part of the paper.

- We intend to continuously update the database as new papers become available. For this reason, the average values reported in this paper may change as more data becomes available.

Table S1 reports comorbidities, demographics, average lab values and average clinical outcomes among all patients, mild patients and severe patients. This expands Table 2 of the paper by including the number of reported cases in each category.

Finally, Table S2 reports statistics on treatments in different continents. The data are reported at a higher level of granularity in early studies in Asia, which hinders direct comparisons. Still, we observe significant differences in the use of hydroxychloroquine and ventilation between Asia, Europe and North America.

2. Supplementary Materials on Clinical Risk Calculators

A. Clinical Characteristics of Study Population. Our mortality cohort comprises 2,859 patients, 739 (25.8%) of whom deceased during hospitalization. The cohort includes patients from both ASST Cremona and HM Hospitals. Table S3 summarizes the clinical characteristics of the cohort, both in aggregate and broken down by survival status. The reported features are those used in the final model, that is age, gender, 3 vitals values, 13 lab results, and 4 comorbidities.

Our infection cohort comprises 3,135 patients, 1,661 (53.0%) of whom tested positive for COVID-19. This cohort only includes patients from ASST Cremona, as negative tests results were not available from HM Hospitals. Table S4 summarizes the clinical characteristics of the cohort, both in aggregate and broken down by test result. Again, the reported features are those used in the final model, that is age, gender, 4 vitals values, and 14 lab values.

B. Method Details. We construct the feature space by aggregating all clinical features for each of the cohorts. We restricted the features to those that have at most 40% of missing values in both datasets (ASST Cremona and HM Hospitals). Missing values are imputed using k-nearest neighbors imputation method (1). The mortality model consists of 22 features. The infection model has a larger feature space, since we are not limited to common features in both datasets. We restrict this model to the 20 most important features, as determined by the algorithm, to ensure usability and reduce the data entry burden on end-users.

We train models for each of the two outcomes of interest (mortality and infection), both including and excluding lab values. This results in a total of four models, referred to as "mortality with lab", "mortality without lab", "infection with lab", and "infection without lab". We use the XGBoost algorithm to train all models (2). We leverage a Bayesian optimization framework to select the best model parameters, using the mean cross-validation area under the curve (AUC) across 40 random seeds as the loss function. This technique results in a more accurate tuning compared to standard grid search, yielding better performance on the test set. We use Scikit-learn (3) to interface XGBoost and Scikit-optimize (4) to perform the hyperparameter tuning. We tune the following parameters for every model: learning rate, γ, λ, α, minimum child weight, maximum tree depth, number of estimators, and the subsample ratio of columns when constructing each tree. All remaining hyperparameters are set to their default value.

[*] https://www.covidanalytics.io/dataset

C. Performance Evaluation. Figure S1a reports the average receiver operating curve and precision-recall curve for each model. The results are averaged across models generated from 40 random seeds. The mortality models have higher average AUCs than the infection models, although the infection models are stronger when evaluated on precision and recall. As expected, predictive performance deteriorates when lab values are excluded. Yet, the models without lab values still achieve strong performance. In particular, the AUC of mortality model drops only moderately when lab values are excluded. Both models see a similar loss in precision/recall when lab values are excluded.

Table S5 reports threshold-based metrics, which evaluate the discriminative ability of the calculators at a fixed cutoff. We ensure a sensitivity of at least 90% to reflect the high cost of false negatives (missing a death or an infection). We select the highest corresponding threshold to maximize specificity. The results show that the accuracy of the models spans 65%–80%. The mortality calculator with lab values achieves a specificity of 76%. The infection model with lab values has lower specificity (63%), but better precision (74% vs. 56%).

Finally, Figure S2 displays calibration plots, showing the true event rates as a function of the average predicted probabilities. The x-axis bins the population by average predicted risk, and the y-axis plots the true event rate (percentage of deaths or infection). All four risk calculators are well calibrated across subgroups, as the fits are close to the 45-degree line. The bottom plot shows the distribution of predicted risk values from the models. For the mortality calculators, the mean predicted values fall below 10% for most samples, whereas the infection calculators distribute the risk more evenly across the cohort. This reflects the fact that mortality is less prevalent than infection.

3. Supplementary Materials on DELPHI-pred and DELPHI-presc

A. Formulation of DELPHI-pred.

A.1. General Formulation. The DELPHI model separates people into 11 possible states:

- **Susceptible (S)**: People who have not been infected.
- **Exposed (E)**: People currently infected, but not contagious and within the incubation period.
- **Infected (I)**: People currently infected and contagious.
- **Undetected (U_R) & (U_D)**: People infected and self-quarantined due to the effects of the disease, but not confirmed due to lack of testing. Some of these people recover (U_R) and some die (U_D).
- **Detected, Hospitalized (DH_R) & (DH_D)**: People who are infected, confirmed, and hospitalized. Some of these people recover (DH_R) and some die (DH_D).
- **Detected, Quarantine (DQ_R) & (DQ_D)**: People who are infected, confirmed, and home-quarantined rather than hospitalized. Some of these people recover (DQ_R) and some die (DQ_D).
- **Recovered (R)**: People who have recovered from the disease (and immune).
- **Deceased (D)**: People who have deceased from the disease.

In addition to main functional states, we introduce helper states to calculate a few useful quantities: Total Hospitalized (TH), Total Detected Deceased (DD) and Total Detected Cases (DT). The full mathematical formulation of the model is as follows:

$$\begin{aligned}
\frac{\mathrm{d}S}{\mathrm{d}t} &= -\widetilde{\alpha}\gamma(t)S(t)I(t)\\
\frac{\mathrm{d}E}{\mathrm{d}t} &= \widetilde{\alpha}\gamma(t)S(t)I(t)\\
\frac{\mathrm{d}I}{\mathrm{d}t} &= r_i E(t) - r_d I(t)\\
\frac{\mathrm{d}U_R}{\mathrm{d}t} &= r_d(1-\widetilde{p}_{dth})(1-p_d)I(t) - r_{ri}U_R(t)\\
\frac{\mathrm{d}DH_R}{\mathrm{d}t} &= r_d(1-\widetilde{p}_{dth})p_d p_h I(t) - r_{rh}DH_R(t)\\
\frac{\mathrm{d}DQ_R}{\mathrm{d}t} &= r_d(1-\widetilde{p}_{dth})p_d(1-p_h)I(t) - r_{ri}DQ_R(t)\\
\frac{\mathrm{d}U_D}{\mathrm{d}t} &= r_d\widetilde{p}_{dth}(1-p_d)I(t) - \widetilde{r}_{dth}U_D(t)\\
\frac{\mathrm{d}DH_D}{\mathrm{d}t} &= r_d\widetilde{p}_{dth}p_d p_h I(t) - \widetilde{r}_{dth}DH_D(t)\\
\frac{\mathrm{d}DQ_D}{\mathrm{d}t} &= r_d\widetilde{p}_{dth}p_d(1-p_h)I(t) - \widetilde{r}_{dth}DQ_D(t)\\
\frac{\mathrm{d}TH}{\mathrm{d}t} &= r_d p_d p_h I(t)\\
\frac{\mathrm{d}DD}{\mathrm{d}t} &= \widetilde{r}_{dth}(DH_D(t) + DQ_D(t))\\
\frac{\mathrm{d}DT}{\mathrm{d}t} &= r_d p_d I(t)\\
\frac{\mathrm{d}R}{\mathrm{d}t} &= r_{ri}(U_R(t) + DQ_R(t)) + r_{rh}DH_R(t)\\
\frac{\mathrm{d}D}{\mathrm{d}t} &= \widetilde{r}_{dth}(U_D(t) + DQ_D(t) + DH_D(t))
\end{aligned}$$

This set of differential equations comprises 11 explicit parameters, defined below. The parameters with a tilde are the parameters that are fitted against historical data for each state; the others are fixed parameters that we estimate using our clinical outcomes database (Section 1).

- $\widetilde{\alpha}$ is the baseline infection rate, constant across all US states.
- $\gamma(t)$ measures the government response and is defined as:

$$\gamma(t) = \frac{2}{\pi} \arctan\left(\frac{-(t - \widetilde{t_0})}{\widetilde{k}}\right) + 1,$$

  where the parameters $\widetilde{t_0}$ and $\widetilde{k}$ capture, respectively, the start date and the strength of the response.
- r_d is the rate of detection. This equals to $\frac{\log 2}{T_d}$, where T_d is the median time to detection (2 days).
- r_i is the rate of infection leaving incubation phase. This equals to $\frac{\log 2}{T_i}$, where T_i is the median time to leave incubation (5 days).
- r_{ri} is the rate of recovery not under hospitalization. This equals to $\frac{\log 2}{T_{ri}}$, where T_{ri} is the median time to recovery when a patient is not under hospitalization (10 days).
- r_{rh} is the rate of recovery under hospitalization. This equals to $\frac{\log 2}{T_{rh}}$, where T_{rh} is the median time to recovery under hospitalization (15 days).
- $\widetilde{r}_{dth}$ is the rate of death.
- $\widetilde{p}_{dth}$ is the mortality rate.
- p_d is the percentage of infection cases detected. This percentage is constant and is set to 20%.
- p_h is the percentage of detected cases hospitalized. This percentage is also constant and set to 15%.

Therefore, we fit on 5 parameters from the list above $(\widetilde{\alpha}, \widetilde{p}_{dth}, \widetilde{r}_{dth}, \widetilde{t_0}, \widetilde{k})$. In addition, we create two additional parameters $\widetilde{k}_1$, $\widetilde{k}_2$ to account for the initial population in the infected (I) and exposed (E) states. We thus fit seven parameters per state.

The parameters are fitted using non-convex optimization methods, including trust-region methods (5) and the Nelder-Mead method (6). We use historical counts of cases and deaths for the fitting procedure. We use a weighted Mean Squared Error (MSE) metric to account for recency and different types of data. The weighted MSE for a training period of T days is defined as:

$$\textbf{Weighted MSE} = \sum_{t=1}^{T} t \cdot \left(DT(t) - \text{Total Detected Cases on Day } t\right)^2 + \lambda^2 \cdot \sum_{t=1}^{T} t \cdot \left(DD(t) - \text{Total Detected Deceased on Day } t\right)^2 .$$

The factor t gives more prominence to more recent data, as recent errors are more likely to propagate into future errors. We set $\lambda = \min\left\{\frac{\text{Total Detected Cases on Day } T}{3 \cdot \text{Total Detected Deceased on Day } T}, 10\right\}$ to balance the fitting between cases and deaths.

A.2. Modeling Government Response. As governments respond to the spread of the epidemic, the rate of infection decreases. We model this by multiplying an initial infection rate with an inverse tangent function, which captures three phases of government response (Figure S3).

- **Phase I**: This phase models the initial response when the government has just started to consider implementing policies. Some people have already changed their behavior in response to early reports, but most people continue business-as-usual activities.
- **Phase II**: This phase is characterized by the sharp decline in infection rate as policies get broadly implemented.
- **Phase III**: This phase reflects the diminishing marginal returns in the decline of the infection rate as the measures reach saturation.

Using parameters $\widetilde{t_0}$ and $\widetilde{k}$, we control the start time and the strength of the measures. We can therefore interpret $\widetilde{t_0}$ as the median day of action, and $\widetilde{k}$ as the median rate of action. This formulation allows us to model, under the same framework, a wide variety of policies—spanning school closures, restriction on mass gatherings, stay-at-home policies, etc.

A.3. DELPHI-pred Validation. Figure S4 shows the projected number of deaths in the United States, with projections made on three different weeks, against historical observations. This complements the corresponding figure in the main text reporting the number of projected vs. actual cases. We see that we were generally able to predict the number of deaths up to 4 weeks ahead with good accuracy. One exception is our prediction made on April 3, which is due to a lack of state-level data on deaths at the time (hence, we had to assume a constant mortality rate per state). But after that, our projections have closely followed historical trends.

B. Formulation of DELPHI-pres.

B.1. Modeling of the Impact of Government Response. Recall that we fit a machine learning model to predict the value of $\gamma(t)$ (fitted by DELPHI-pred), as a function of the policies in place. The objective is to evaluate the impact of each policy on the infection rate in order to simulate its overall effect on the dynamics of the pandemic. Figure S5 shows the resulting regression tree, using state-level data in the United States. The results show that more stringent policies result in lower values of $\gamma(t)$, hence in lower infection rates. For instance, in states with no measure in place, the predicted value of $\gamma(t)$ is 1.304; in states where a stay-at-home policy is in place, the predicted value of $\gamma(t)$ is 0.312; in states where partial social distancing policies are in place, the predicted value of $\gamma(t)$ falls in between.

The main objective of DELPHI-presc is to modify the value of $\gamma(t)$ in DELPHI-pred to account for future changes in social distancing policies, using the values predicted by the tree shown in Figure S5. To this end, we define the following quantities:

- t_c is the time of the policy change.

- k_0 is a *normalized* pair-wise difference between the predicted values of $\gamma(t)$ between policies (with respect to the largest predicted value of $\gamma(t)$ under no measure). For instance, transitioning from stay-at-home to no measure induces an offset $(1.304 - 0.312)/1.304 = +0.761$. All values can be found in Table S6—the offset is positive if the new policy is more lenient, and negative if it is more stringent.
- p_0 is the normalized value of the current policy.

We then correct the government response as follows:

$$\gamma'(t) = \max\left\{\frac{2}{\pi}\arctan\left(-\frac{t-\widetilde{t_0}}{k}\right) + 1 + k_0 \cdot \min\left[\frac{2-\gamma(t_c)}{1-p_0}, \frac{\gamma(t_c)}{p_0}\right], 0\right\}, \quad \forall t \geq t_c.$$

For example, if we are currently in Lockdown and are moving to No measure, then we obtain $k_0 = 0.787$, $p_0 = 0.329/1.544 = 0.213$ and $\gamma(t_c) = \frac{2}{\pi}\arctan\left(-\frac{t_c-\widetilde{t_0}}{k}\right) + 1$.

B.2. Application to Policy Assessment. To assess any policy, we run the DELPHI-pred model (governed by the system of differential equations), using the value of the infection rate derived in Section Eq. (B.1). We report the impact of the different policies on the case count in the main body of the paper. Figure S6 provides a similar visualization of the effect on the death count in the state of New York. We can draw similar observations regarding the impact of the various policies and the impact of the timing of these policies.

4. Supplementary Materials on Ventilator Allocation

We now detail the formulation of the optimization model proposed for ventilator allocation. We begin by specifying the model mathematically, then discuss data sources and parameter calibration.

A. Formulation. We consider S states, indexed by $s = 1, \cdots, S$, and D days, indexed by $d = 1, \cdots, D$.

Data. In formulating the problem, we consider the following data as given:
- $v_{s,d}$ is the demand for ventilators in state s on day d.
- b_s is the base supply of ventilators starting in each state s.
- n_d is the surge supply of ventilators distributed by the federal government on day d.
- $d_{s,s'}$ is the distance between state s and state s'.
- $\tau_{s,s'}$ is the lead time between state s and state s'.

We note two comments regarding these inputs. First, the surge supply n_d corresponds to the number of ventilators that are actually distributed by the federal government on day d: the details of managing the federal stockpile fall beyond the scope of this model. More generally, n_d represents supply available from any exogenous, centralized source. Second, we consider distances between states as a way to encourage transfers between neighboring states. We also calibrate the distances such that $d_{s,s} > 0$ for each state s, to ensure we do not propose meaningless transfers from state s to itself.

Decisions. We define integer decision variables as follows:
- $x_{s,d} \in \mathbb{Z}^+$ is the supply of ventilators in state s on day d.
- $y_{s,s',d} \in \mathbb{Z}^+$ is the number of ventilators sent from state s to state s' on day d.
- $z_{s,d} \in \mathbb{Z}^+$ is the additional supply state s receives from the federal government on day d.
- $w_{s,d} \in \mathbb{Z}^+$ is the shortage of ventilators in state s on day d relative to the demand $v_{s,d}$.
- $\Delta_{s,d} \in \mathbb{Z}^+$ is the shortage of ventilators in state s on day d relative to the demand with a buffer.

Parameters. We define the following parameters, which control different policy trade-offs:
- $f_{\max} \in [0,1]$ is the maximum fraction of its base supply that each state is willing to share. A value of $f_{\max} = 0$ indicates that states are not willing to share any ventilator with other states; a value of $f_{\max} = 1$ indicates that states are willing to share all their ventilator supply with other states.
- $\alpha \in [0, \infty)$ is the percentage of projected demand that states would like to plan for with a supply buffer. For example, $\alpha = 0.1$ will penalize any solution such that supply falls within 10% of projected demand.
- $\lambda \in [0, \infty)$ is a regularization parameter that captures the trade-off between the financial and logistical cost of interstate transfers with the public health cost of ventilator shortages.
- $t_{\min} \in \mathbb{Z}^+$ is the number of days a ventilator is in use after it is shipped to a new location, allowing to control for excessive transfers.
- $\rho \in [0,1]$ is a relative cost parameter capturing the relative importance of projected shortages vs. worst-case shortages. Each unit of supply that falls short of the projected demand is assigned a cost of 1. Each unit of supply that exceeds the demand but does not exceed the state's desired supply buffer is assigned a cost of ρ.

Objective. The problem of allocating scarce resources in a pandemic is complex because of the necessity to balance competing interests. We identify two key operational goals: improving public health outcomes, and reducing financial cost. We therefore formulate the ventilator allocation problem with two objectives, minimizing total shortage costs as well as total ventilator transfer costs. Each unit of ventilator shortage is assigned a weight of 1 (for shortage relative to the projected demand) or a weight of $\rho \leq 1$ (for shortage relative to the buffered demand). We formalize the bi-objective problem by means of a penalty on transfers, weighted with a trade-off parameter λ.

$$\min \quad \sum_{s=1}^{S}\sum_{d=1}^{D}(w_{s,d} + \rho\Delta_{s,d}) + \lambda\sum_{s=1}^{S}\sum_{s'=1}^{S}\sum_{d=1}^{D} d_{s,s'}y_{s,s',d}. \qquad [1a]$$

Note that ventilators distributed by the federal government are not penalized, as our model simply treats this source as exogenous.

Constraints.

- Initial supply for each state s:

$$x_{s,0} = b_s, \quad \forall s = 1, \cdots, S. \tag{1b}$$

- The supply in each state s on each day d remains higher than the fraction of its initial supply the state wants to retain:

$$x_{s,d} \geq (1 - f_{\max})b_s, \quad \forall s = 1, \cdots, S, \ d = 1, \cdots, D. \tag{1c}$$

- The transfers from the federal government cannot exceed the available surge supply on each day d:

$$\sum_{s=1}^{S} z_{s,d} \leq n_d, \quad \forall d = 1, \cdots, D. \tag{1d}$$

- The shortage variable corresponds to the positive part of the difference between ventilator demand and supply, if positive, for each state s and day d:

$$w_{s,d} \geq v_{s,d} - x_{s,d}, \quad \forall s = 1, \cdots, S, \ d = 1, \cdots, D. \tag{1e}$$

- The buffer shortage variable is defined such that the total (actual plus buffer) shortage corresponds to the difference between buffered demand and ventilator supply, if positive, for each state s and day d:

$$w_{s,d} + \Delta_{s,d} \geq (1 + \alpha)v_{s,d} - x_{s,d}, \quad \forall s = 1, \cdots, S, \ d = 1, \cdots, D. \tag{1f}$$

- (Conservation of flow) For each state s and day d, today's supply is equal to yesterday's supply plus what is received today from the government and the other states, minus what is sent to other states, with $\tau_{s',s}$ reflecting shipments' lead times:

$$x_{s,d} = x_{s,d-1} + z_{s,d} + \sum_{s'=1}^{S} y_{s',s,d-\tau_{s',s}} - \sum_{s'=1}^{S} y_{s,s',d}, \quad \forall s = 1, \cdots, S, \ d = 1, \cdots, D. \tag{1g}$$

- (Minimum days in use) For each state s and day d, any incoming ventilator, either from another state or from the federal government, cannot be shipped out for at least t_{min} days. This constraint ensures that ventilators are not transferred too often.

$$\sum_{d'=\max(1,d-t_{\min})}^{d-1} \left(z_{s,d'} + \sum_{s'=1}^{S} y_{s',s,d'} \right) \leq x_{s,d}, \quad \forall s = 1, \cdots, S, \ d = 1, \cdots, D. \tag{1h}$$

- Any state s facing a shortage on day d cannot ship any ventilators to other states on day d. To write this constraint, we define auxiliary binary variables $a_{s,d} \in \{0, 1\}$ indicating if there is a shortage in state s on day d and a parameter $V_{\max}$ providing a trivial upper bound on the number of ventilators a state can ship per day (we use a value of 3,000 which does not restrict the solution, as it exceeds the shortage faced by any state on any given day).

$$w_{s,d} + \Delta_{s,d} \leq v_{s,d}(1 + \alpha)a_{s,d}, \tag{1i}$$

$$\sum_{s'=1}^{S} y_{s,s',d} \leq V_{\max}(1 - a_{s,d}). \tag{1j}$$

B. Data Sources and Parameter Calibration. Our optimization model is complex enough to model high-level dynamics of scarce resource allocation, yet simple enough to only require simple data inputs. We now describe our methodology in collecting the key data necessary to solve this optimization problem.

Demand. The most important input data is the forecasted ventilator demand $v_{s,d}$. Consistent with our end-to-end data-driven approach, and in contrast with other ventilator allocation approaches (7), we develop our own demand forecasts using DELPHI-pred (Section 3 of the main text). Recall that DELPHI-pred does not only estimate the number of cases, but also the number of hospitalizations, equal to $DH_R + DH_D$. We then apply a 25% ratio to estimate the number of ventilators in use—given that, in our clinical outcomes database, 25% of hospitalized patients are on a ventilator. Ultimately, we can use the DELPHI-pred outputs to derive projections of ventilator demand at the state level and at the daily level—consistently with the optimization input $v_{s,d}$.

Supply. It can be difficult to estimate how many ventilators are available in each state as well as at the federal level. For the base supply b_s, we use inventory levels from a 2010 American Medical Association report (8). We adjust this number for population growth, under the assumption that the number of ventilators per capita has remained constant in each state.

Of course, ventilators can also be used to treat non-COVID-19 patients. We assume that 50% of ventilator supply across the board is unavailable due to non-COVID-19 usage, in line with other estimates (7).

In addition, our model takes into account the daily availability n_d of ventilators at the federal level. Estimating this quantity from publicly-available sources is both difficult due to limited data and politically fraught. We use the estimate from the Society for Critical Medicine that the federal stockpile contains at least 12,700 ventilators (9). Some news reports suggest a lower estimate of 10,000 based on some defects in the stockpiled equipment (10), while others suggest an estimate of 16,600 based on older model repairs (11). Based on these reference points, we estimate roughly 13,500 available ventilators and assume that they can be deployed evenly over a month. In other words, we allow 450 ventilators to be deployed each day for 30 days (starting on day 4 to allow for lead times). This gradual release reflects potential operational constraints and strategic considerations of controlling the release of inventory in case of unexpected outbreaks. Yet, given the underlying uncertainty, we perform sensitivity analysis to explore how the model's recommendation varies with the federal stockpile.

Distances and lead times. We compute the interstate distance $d_{s,s'}$ as the Euclidean distance between the centers of states s and s', and we let the lead time parameter $\tau_{s,s'}$ equal 3 days for every pair of states. Our choice of a conservative uniform lead time for shipments is motivated by simplicity concerns. This could be improved, in future work, to better reflect efficiencies in the US shipping infrastructure.

Trade-off parameters.

- Understanding the impact of states' willingness to share ventilators with other states is a key takeaway from our model. In Figure 7B, we vary the fraction $f_{\max}$ of each state's pooling supply between $\{0\%, 5\%, 10\%, 15\%, 20\%\}$. Results indicate there is little additional efficiency to be gained from states sharing more than 20% of their supply.
- Understanding the relative importance of federal surge supply compared to interstate transfers is another interesting takeaway from our model. In Figure 7A, we show the effects of removing federal surge supply, or preventing interstate transfers, on ventilator shortage reduction. We show more detailed sensitivity analysis results in Section C.
- We vary the parameter λ to derive the the Pareto-optimal frontier of the trade-off between inter-state transfers vs. ventilator shortages (for instance in Fig. 7B). As λ tends to zero, transfers incur no cost other than rendering the ventilators unavailable during shipment; as λ tends to infinity, transfers become heavily discouraged.
- The parameter α models uncertainty in the demand forecast as well as robustness to inefficiency in ventilator allocation within each state. We vary the percentage α of buffered demand within $\{0\%, 5\%, 10\%, 20\%\}$ in Section C.

Finally, we choose the following values for the remaining two parameters, which have a small impact on the final solution.

- We set the value of $t_{\min}$ to 10 days, based on the clinical outcomes database (Section 1).
- We set ρ, the cost of shortage with respect to buffered demand relative to the cost of shortage with respect to real demand, to 0.25.

C. Sensitivity analysis. In the main text, we discuss the impact of the federal surge and inter-state coordination on alleviating ventilator shortages, showing that ventilator shortages can be eliminated through limited transfers among states and from the federal government. The results also suggest trade-offs between the number of inter-state transfers, the amount of ventilator shortages, and the fraction of ventilators that each state is willing to share (captured by the $f_{\max}$ parameter). We report additional results to underscore the trade-offs between inter-state transfers, the ventilator supply from the federal government, and the robustness of the solution—that is, the exposure of the states to ventilator shortages if demand exceeds our projections.

To this end, Figure S7 shows the Pareto-optimal frontier between the model's two objectives, inter-state transfer distance and ventilator shortage, as a function of two model parameters: α (capturing the demand buffer that states would like to plan for) and a *surge supply multiplier* (capturing by how much the federal government's ventilator supply varies from our estimates). Note that the buffer α does not impact the number of inter-state transfers and the amount of ventilator shortages too significantly—suggesting that the solution can be made robust at limited overall costs. In contrast, as the surge supply is decreased, the number of required ventilator transfers and the amount of ventilator shortages increase—highlighting the need for stronger cooperation between states as federal supply drops.

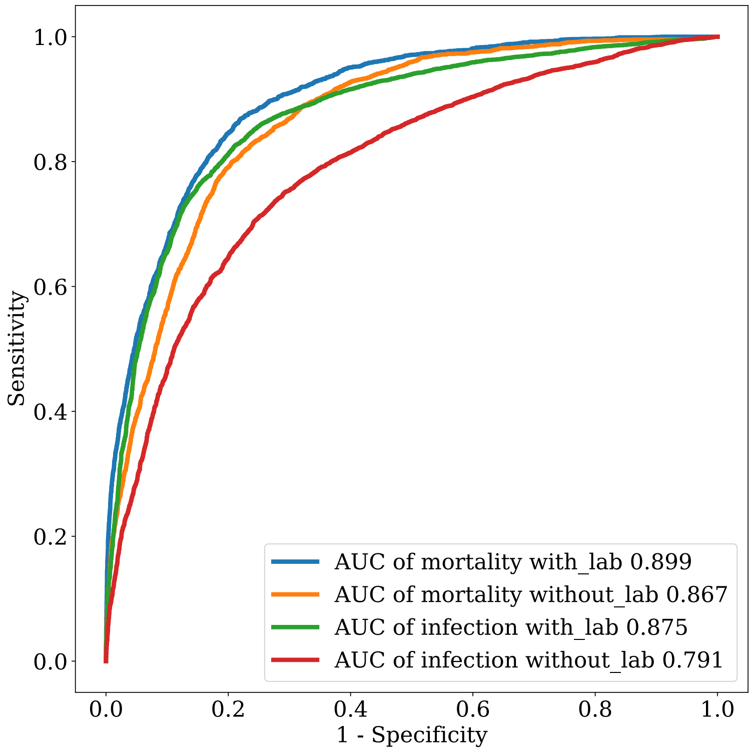

(a) Average ROC Curves

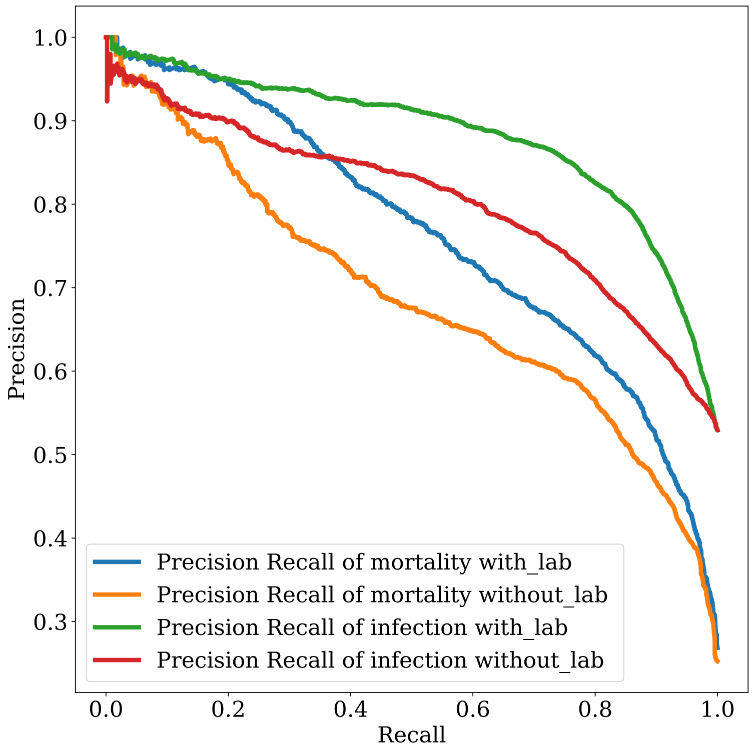

(b) Average Precision Recall Curves

Fig. S1. Bootstrapped results on ROC and Precision Recall curves for all calculators on the testing set.

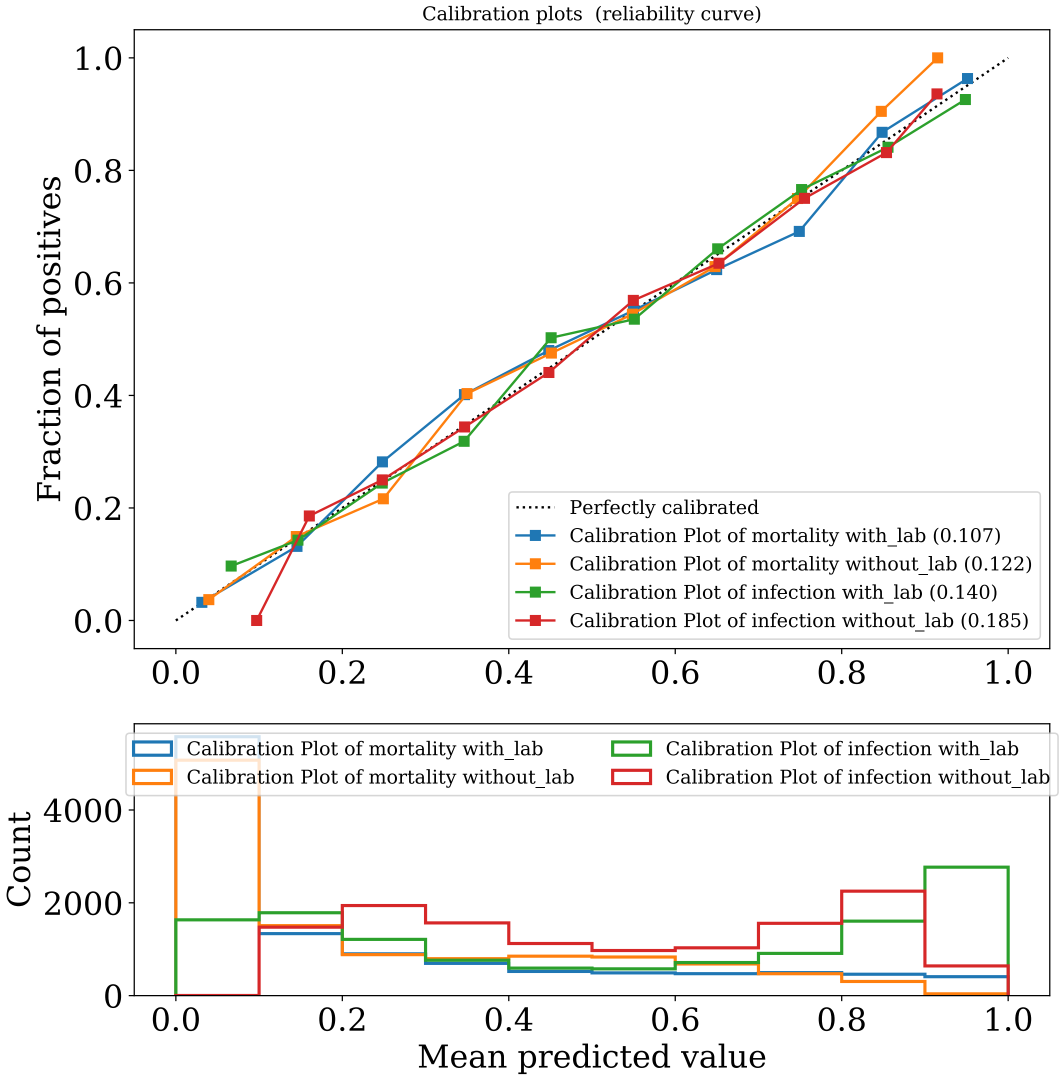

Fig. S2. Bootstrapped Results on the Calibration Curves for both risk calculators on the testing set. The intervals are: [0,10%], (10,20%], (20,30%], …, (90,100%]. The event rates are plotted against the bin mid-points. An ideal event rate is marked by the dotted 45 degree line.

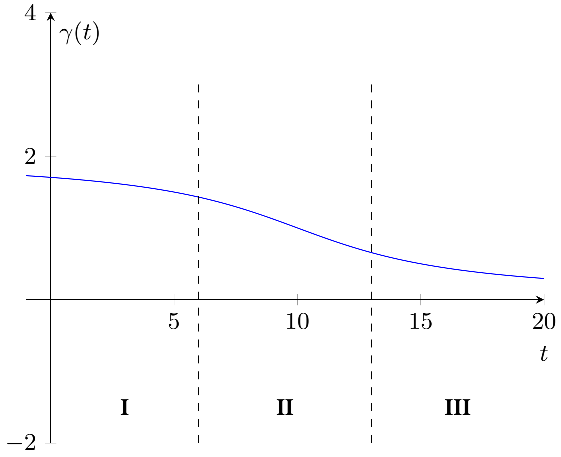

Fig. S3. Illustration of $\gamma(t) = \frac{2}{\pi}\arctan(-\frac{t-10}{5}) + 1$ (i.e., $\widetilde{t_0} = 10$ and $k = 5$).

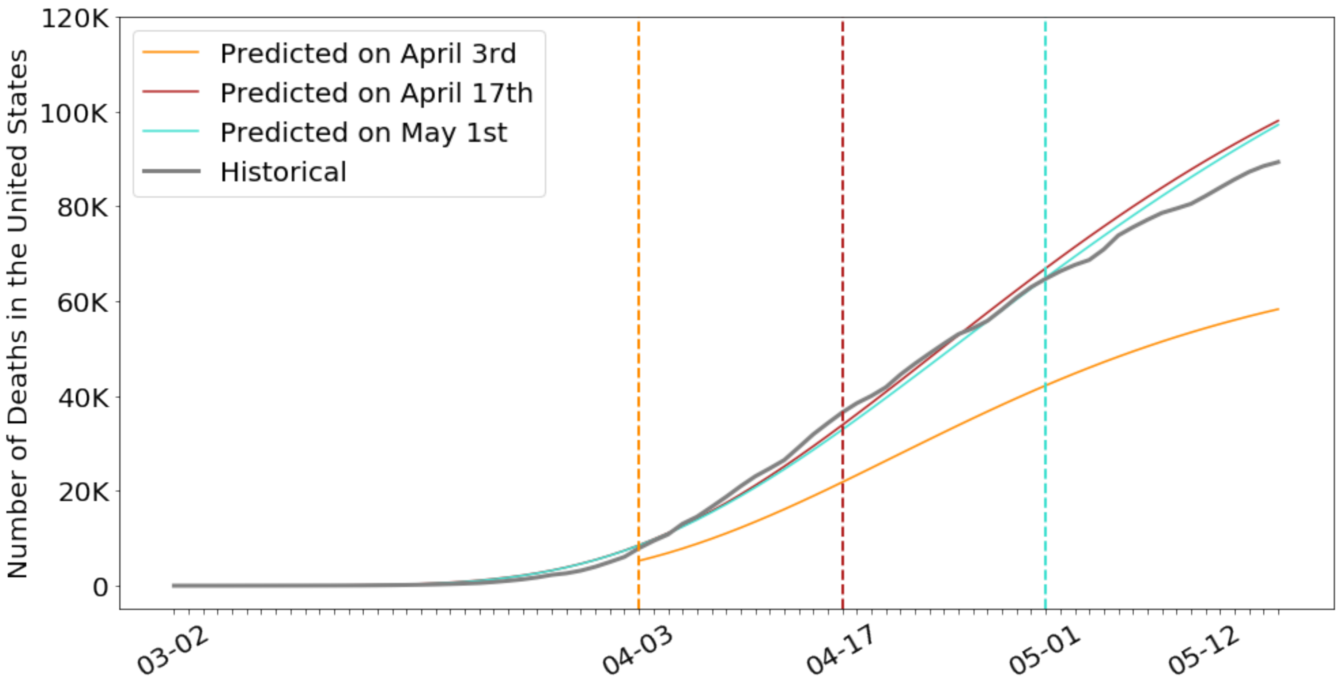

Fig. S4. Cumulative number of deaths in the United States according to our projections made at different points in time, against actual observations

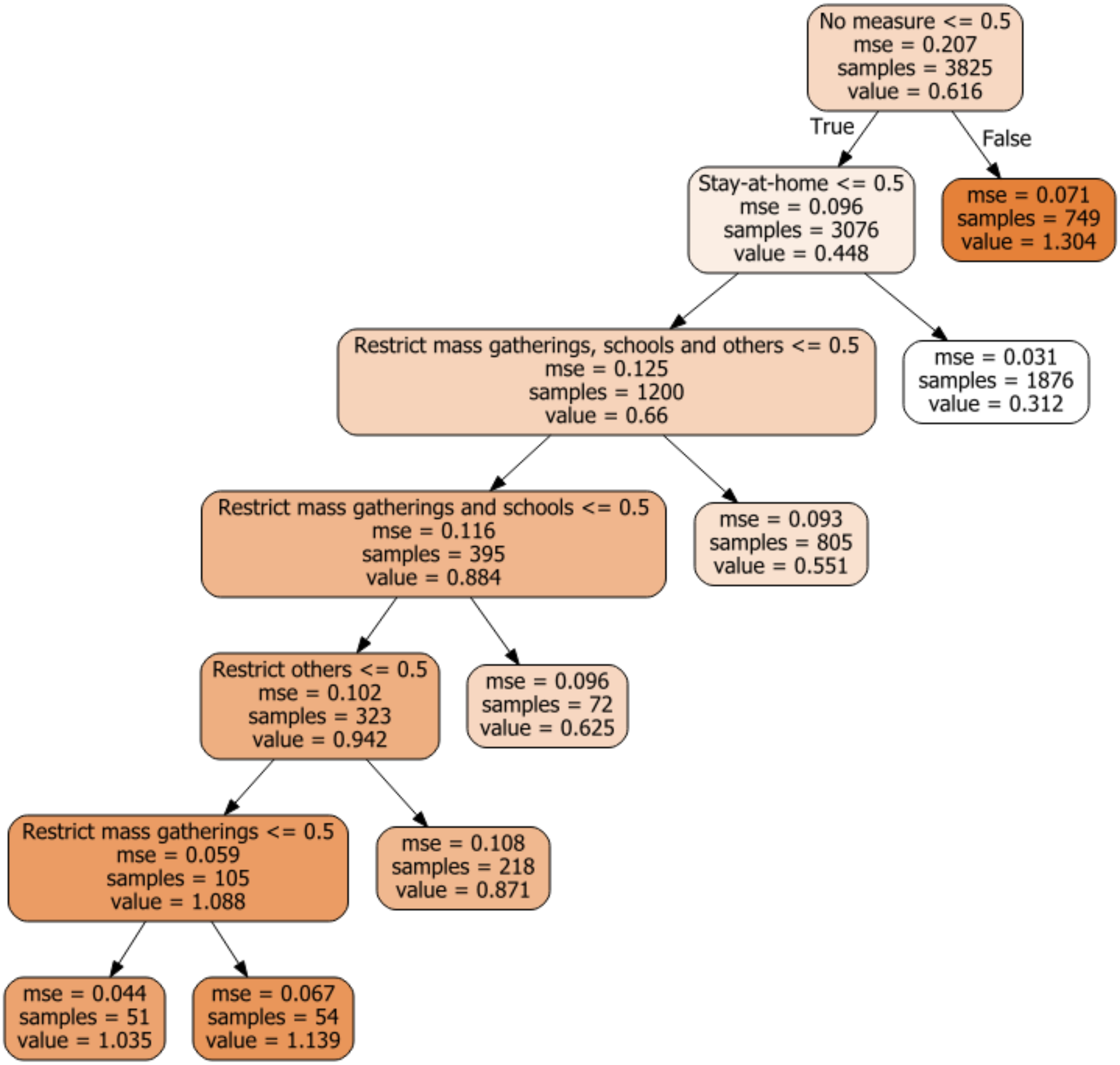

Fig. S5. Regression Tree (CART) predicting an average value of $\gamma(t)$ for each policy.

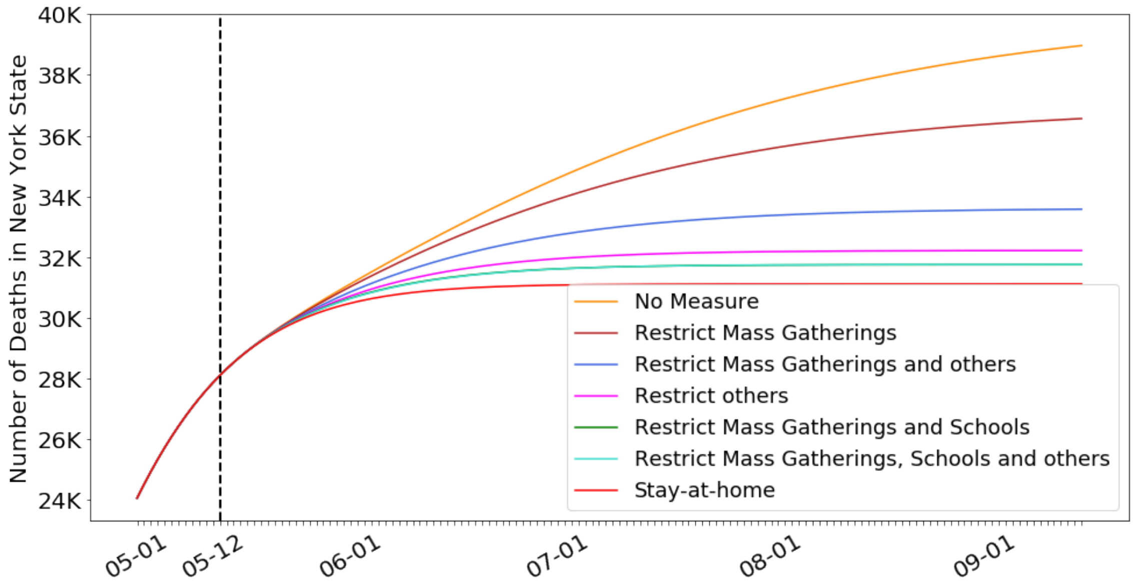

(a) Impact of different policies on the future number of deaths, in NY

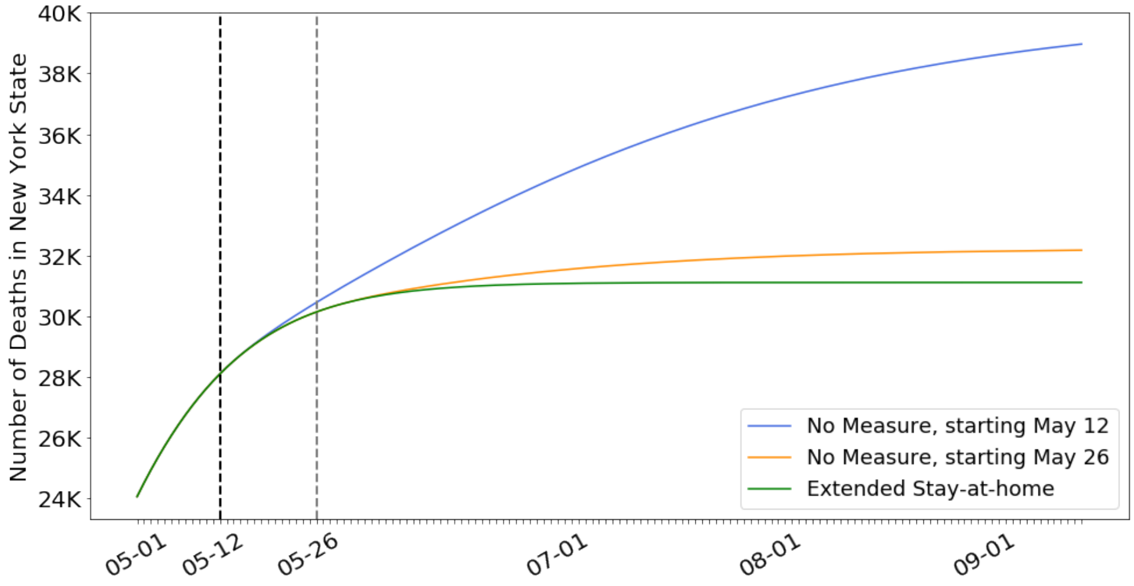

(b) Impact of the timing of policies on the future number of deaths, in NY.

Fig. S6. Impact of different policies on the future number of deaths in the State of New York, for different policies and policy start dates.

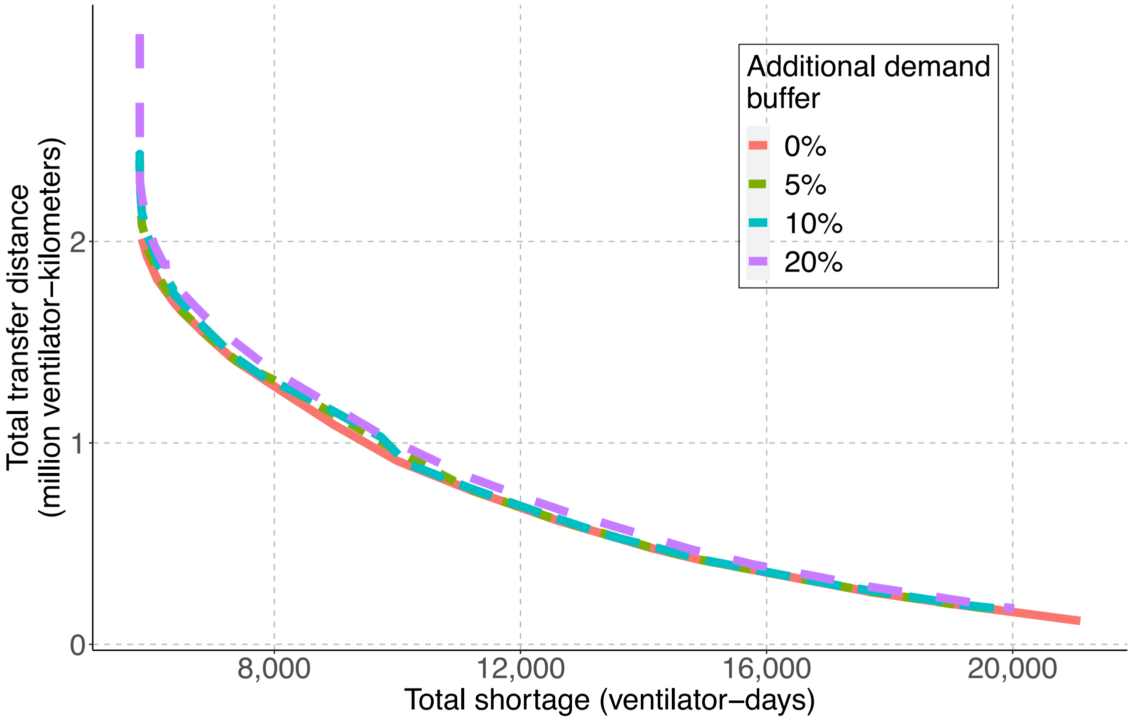

(a) Impact of additional buffer

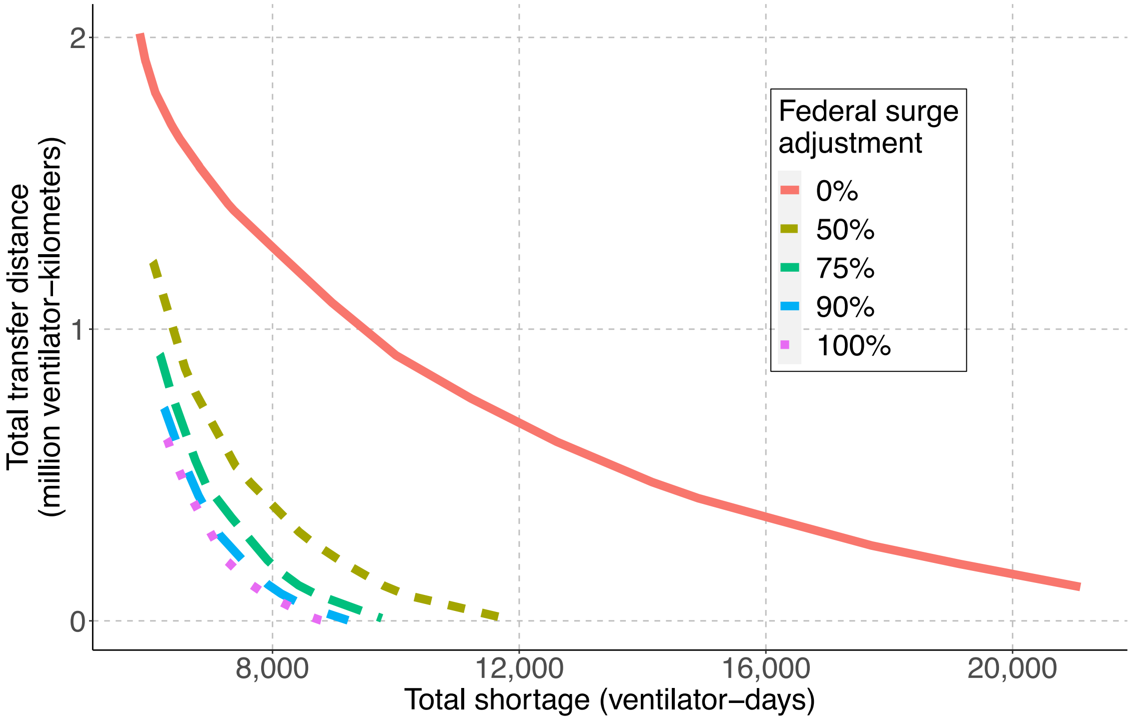

(b) Impact of federal surge

Fig. S7. Influence of additional buffer and federal surge availability on ventilator shortage and ventilator transfers.

Table S1. Comorbidities, demographics, average lab values, average length of stay and projected mortality among COVID-19 patients, in aggregate and broken down into mild/severe patients.

Feature	All Patients		Mild Patients		Severe Patients	
	No. Report	Avg.	No. Report.	Avg.	No. Report.	Avg.
Demographics						
Male (%)	131,200	53.0%	9,570	48.8%	10,120	68.7%
Age (years)	119,000	51.3	8,022	46.1	9,685	68.2
White/European (%)	55,490	22.2%	10,120	9.7%	9,887	63.9%
African American (%)	55,490	5.4%	10,120	3.5%	9,887	2.5%
Asian (%)	55,320	51.3%	10,300	80.2%	9,933	31.2%
Hispanic/Latino	50,630	19.9%	8,017	0%	9,107	0%
Multiple ethnicities/other	55,190	3.6%	10,120	6.9%	9,887	2.7%
Comorbidities						
Smoking history	27,900	16.1%	6,080	12.2%	1,973	16.6%
Hypertension	38,390	35.9%	8,252	15.2%	8,449	54.4%
Diabetes	39,790	20.8%	8,396	6.8%	8,818	26.1%
Cardio Disease	40,030	12.4%	8,028	3.0%	9,540	20.3%
COPD	34,150	6.0%	6,297	2.8%	8,727	10.0%
Cancer	29,170	7.2%	6,259	3.2%	8,355	12.9%
Liver Disease	18,300	2.8%	1,875	2.3%	6,832	3.5%
Cebrovascular	6,830	9.8%	3,245	2.7%	1,360	24.8%
Kidney Disease	35,500	5.7%	6,152	1.2%	8,139	10.8%
Lab values						
WBC Count (10^9/L)	19,970	6.41	5,403	5.07	2,305	6.80
Neutrophil Count (10^9/L)	12,500	4.72	2,236	5.12	1,410	5.78
Platelet Count (10^9/L)	12,125	195.7	5,165	184.0	2,105	170.4
ALT (U/L)	14,467	29.0	2,840	24.6	2,428	31.1
AST (U/L)	14,214	37.3	2,766	27.1	2,366	45.7
BUN (mmol/L)	4,822	5.22	1,700	4.18	1,138	6.86
Creatinine (μmol/L)	8,504	63.08	2,529	66.0	2,454	56.4
CRP Count (mg/L)	17,090	76.5	2,573	18.9	2,339	94.1
Interleukin-6 (pg/mL)	2,582	24.57	1,127	4.17	552	38.63
Procalcitonin (ng/mL)	14,750	2.26	1,468	1.85	1,969	4.81
D-Dimer (mg/L)	13,330	38.81	2,478	8.04	2,401	165.9
Length of Stay (days)	16,010	10.7	4,131	14.0	5,642	7.97
Proj. Mortality (%)	111,700	11.7%	7,428	0.4%	9,146	74.0%

Table S2. Count and prevalence of treatments among COVID-19 patients, broken down per continent (Asia, Europe, North America). A "-" indicates that fewer than 100 patients in a subpopulation reported on this symptom.

Treatment	Asia		Europe		North America	
	No. Report	Prev. (%)	No. Report.	Prev. (%)	No. Report.	Prev. (%)
Kaletra	5,665	35.2%	–	–	–	–
Oseltamivir	5,901	25.1%	–	–	–	–
Remdesivir	337	47.4%	–	–	868	10.3%
Arbidol	5,902	34.8%	–	–	–	–
Interferon	3,647	51.8%	–	–	–	–
Hydroxychloroquine	6,008	0.7%	–	–	1,235	61.7%
Invasive Ventilation	7,945	8.0%	75,120	4.8%	5,840	19.3%
Proj. Mortality	12,820	16.7%	79,750	9.9%	19,060	15.8%

Table S3. Characteristics of study population for mortality prediction model.

	All ($N = 2,831$)	Survivors ($N = 2,120$)	Non-Survivors ($N = 711$)	P-Value
Age	68.0 (57.0-79.0)	63.0 (54.0-74.0)	81.0 (73.2-86.0)	1.28E-185
Female *	1095.0 (38.7%)	868.0 (40.9%)	227.0 (31.9%)	1.18E-05
Heart Rate	89.0 (79.0-101.0)	90.0 (80.0-102.0)	87.0 (78.0-100.0)	1.29E-03
Oxygen Saturation	94.0 (90.0-96.0)	94.4 (92.0-96.0)	88.5 (80.0-93.6)	3.16E-37
Temperature (F)	98.4 (97.5-99.7)	98.4 (97.5-99.6)	98.8 (97.7-100.0)	2.42E-04
Alanine Aminotransferase	27.0 (17.0-44.0)	27.8 (17.5-45.0)	25.5 (16.0-41.0)	3.77E-02
Aspartate Aminotransferase	36.0 (25.0-55.0)	34.0 (24.4-51.0)	45.0 (30.0-69.0)	1.55E-11
Blood Glucose	118.0 (105.0-141.0)	115.0 (103.4-133.0)	134.0 (113.0-171.0)	1.12E-22
Blood Urea Nitrogen	17.0 (12.6-25.2)	15.0 (11.5-20.0)	29.5 (20.3-47.2)	1.02E-65
C-Reactive Protein	74.2 (29.1-149.5)	58.6 (22.7-119.3)	141.1 (72.0-223.1)	4.76E-50
Creatinine	1.0 (0.8-1.2)	0.9 (0.7-1.1)	1.3 (1.0-1.8)	2.84E-36
Hemoglobin	13.9 (12.7-15.0)	14.0 (12.9-15.0)	13.5 (12.0-14.7)	9.11E-10
Mean Corpsular Volume	87.8 (84.9-91.0)	87.5 (84.7-90.4)	89.3 (85.8-92.7)	2.80E-08
Platelets	201.0 (156.0-263.0)	206.0 (160.0-266.5)	185.0 (141.0-246.8)	6.62E-08
Potassium	4.1 (3.7-4.4)	4.0 (3.7-4.4)	4.1 (3.7-4.6)	1.43E-04
Prothrombin Time (INR)	1.1 (1.0-1.2)	1.1 (1.0-1.2)	1.1 (1.0-1.3)	3.20E-05
Sodium	137.1 (135.0-140.0)	137.0 (135.0-139.4)	138.0 (135.0-141.0)	5.65E-08
White Blood Cell Count	6.8 (5.2-9.2)	6.5 (5.0-8.7)	8.0 (5.7-11.4)	3.00E-15
Cardiac dysrhythmias *	200.0 (7.1%)	127.0 (6.0%)	73.0 (10.3%)	6.50E-04
Chronic kidney disease *	65.0 (2.3%)	33.0 (1.6%)	32.0 (4.5%)	3.67E-04
Heart disease *	125.0 (4.4%)	80.0 (3.8%)	45.0 (6.3%)	1.10E-02
Diabetes *	345.0 (12.2%)	234.0 (11.0%)	111.0 (15.6%)	2.73E-03
Mortality *	711 (25.1%)	0 (0%)	711 (100%)	–

* Count (proportion) is reported for binary variables.

Table S4. Characteristics of study population for infection test prediction model.

	All ($N = 3,135$)	No Infection ($N = 1,474$)	Infection ($N = 1,661$)	P-Value
Age	63.0 (49.0-78.0)	58.0 (42.0-78.0)	66.0 (55.0-78.5)	9.00E-28
Female *	1444.0 (46.1%)	777.0 (52.7%)	667.0 (40.2%)	1.70E-12
Heart Rate	88.5 (78.0-100.5)	89.0 (78.0-101.2)	88.0 (78.2-100.0)	6.13E-01
Oxygen Saturation	95.4 (91.8-97.0)	96.5 (94.8-97.5)	94.2 (89.6-96.4)	1.68E-31
Respiratory Frequency	18.0 (16.0-19.0)	18.0 (16.0-18.0)	18.0 (16.0-20.0)	9.64E-21
Temperature	98.3 (97.5-99.5)	97.7 (97.2-98.7)	99.0 (97.9-100.0)	4.51E-80
Alanine Aminotransferase	22.0 (15.0-37.0)	19.0 (13.0-30.0)	27.0 (18.0-43.0)	6.59E-09
Aspartate Aminotransferase	29.0 (21.0-47.0)	23.0 (19.0-31.0)	37.0 (26.0-57.0)	1.20E-20
Blood Urea Nitrogen	17.0 (13.0-25.0)	16.0 (12.0-22.0)	18.0 (13.0-27.0)	3.78E-05
Calcium	9.3 (8.9-9.7)	9.6 (9.2-9.9)	9.0 (8.7-9.4)	1.90E-96
C-Reactive Protein	31.0 (3.4-107.6)	4.7 (1.1-35.4)	69.8 (23.2-152.3)	1.28E-83
Creatinine	0.9 (0.8-1.2)	0.9 (0.7-1.1)	1.0 (0.8-1.2)	2.19E-05
Hemoglobin	13.5 (12.3-14.7)	13.4 (12.1-14.6)	13.6 (12.5-14.8)	5.70E-05
Mean Corpsular Volume	87.2 (84.0-90.3)	87.7 (84.2-90.7)	86.8 (83.9-90.0)	1.43E-01
Platelets	223.0 (174.0-285.0)	241.0 (198.0-297.0)	202.0 (156.0-266.0)	1.41E-18
Red Cell Distrbution Width	13.2 (12.5-14.3)	13.2 (12.5-14.5)	13.1 (12.4-14.0)	1.59E-06
Sodium	139.0 (137.0-141.0)	140.0 (138.0-142.0)	139.0 (136.0-141.0)	1.12E-14
Prothrombin Time (INR)	1.0 (1.0-1.1)	1.1 (1.0-1.1)	1.0 (1.0-1.1)	8.96E-01
Total Bilirubin	0.6 (0.5-0.8)	0.6 (0.4-0.9)	0.6 (0.5-0.8)	8.83E-03
White Blood Cell Count	7.6 (5.8-10.1)	8.7 (7.0-11.1)	6.6 (5.1-8.7)	7.59E-38
COVID-19 Positive Test *	1661 (53.0%)	0 (0%)	1661 (100%)	–

* Count (proportion) is reported for binary variables.

Table S5. Performance evaluation summary. Average results across 40 random seeds are reported along with 95% confidence intervals. A minimum threshold of 90% sensitivity is enforced.

Model Type	Lab Values	Threshold	Accuracy	Sensitivity	Specificity	Precision	Negative predictive value	False positive rate
**Mortality**	Present	17.45 (15.61,19.29)	79.29 (77.47,81.11)	90.38 (90.38,90.38)	75.66 (73.25,78.07)	55.86 (53.46,58.25)	95.97 (95.84,96.1)	24.34 (21.93,26.75)
**Mortality**	Absent	12.66 (11.11,14.2)	70.85 (68.45,73.24)	90.38 (90.38,90.38)	64.53 (61.37,67.7)	46.15 (43.96,48.33)	95.3 (95.04,95.55)	35.47 (32.3,38.63)
**Infection**	Present	28.32 (26.63,30.02)	77.58 (76.48,78.68)	90.36 (90.36,90.36)	63.24 (60.9,65.58)	73.59 (72.33,74.85)	85.26 (84.79,85.73)	36.76 (34.42,39.1)
**Infection**	Absent	27.51 (26.55,28.47)	66.31 (65.37,67.25)	90.36 (90.36,90.36)	39.32 (37.33,41.32)	62.64 (61.85,63.44)	78.12 (77.28,78.96)	60.68 (58.68,62.67)

Table S6. Values of normalized offset computations to correct the estimation of $\gamma(t)$. Policies include: (i) *No measure* ("none"); (ii) *Restrict mass gatherings* ("R-MG"); (iii) *Restrict others* ("R-O"); (iv) *Authorize schools, restrict mass gatherings and others* ("R-MG-O"); (v) *Restrict mass gatherings and schools* ("R-MG-S"); (vi) *Restrict mass gatherings, schools and others* ("R-MG-S-O"); and (vii) *Stay-at-home* ("SAH").

from/to	none	R-MG	R-O	R-MG-O	R-MG-S	R-MG-S-O	SAH
none	0	-0.127	-0.332	-0.206	-0.521	-0.577	-0.761
R-MG	+0.127	0	-0.206	-0.080	-0.294	-0.451	-0.634
R-O	+0.332	+0.206	0	+0.126	-0.189	-0.245	-0.429
R-MG-O	+0.206	+0.080	-0.126	0	-0.314	-0.371	-0.554
R-MG-S	+0.521	+0.294	+0.189	+0.314	0	-0.057	-0.240
R-MG-S-O	+0.577	+0.451	+0.245	+0.371	+0.057	0	-0.183
SAH	+0.761	+0.634	+0.429	+0.554	+0.240	+0.183	0

Bibliography

1. O Troyanskaya, et al., Missing value estimation methods for dna microarrays. *Bioinformatics* **17**, 520–525 (2001).
2. T Chen, C Guestrin, XGBoost: A scalable tree boosting system in *Proceedings of the 22nd ACM SIGKDD International Conference on Knowledge Discovery and Data Mining*, KDD '16. (ACM, New York, NY, USA), pp. 785–794 (2016).
3. F Pedregosa, et al., Scikit-learn: Machine learning in Python. *J. machine Learn. research* **12**, 2825–2830 (2011).
4. T Head, et al., Scikit-optimize (scikit-optimize/scikit-optimize: v0.5.2) (2017).
5. RH Byrd, JC Gilbert, J Nocedal, A trust region method based on interior point techniques for nonlinear programming. *Math. programming* **89**, 149–185 (2000).
6. JC Lagarias, JA Reeds, MH Wright, PE Wright, Convergence properties of the nelder–mead simplex method in low dimensions. *SIAM J. on optimization* **9**, 112–147 (1998).
7. S Mehrotra, H Rahimian, M Barah, F Luo, K Schantz, A model of supply-chain decisions for resource sharing with an application to ventilator allocation to combat COVID-19. *Nav. Res. Logist. (NRL)* (2020).
8. L Rubinson, et al., Mechanical ventilators in us acute care hospitals. *Disaster medicine public health preparedness* **4**, 199–206 (2010).
9. NA Halpern, KS Tan, VT SCCM, Us icu resource availability for covid-19. *Soc. Critical Care Medicine, March* **25** (2020).
10. New York Times, A Ventilator Stockpile, With One Hitch: Thousands Do Not Work (https://www.nytimes.com/2020/04/01/us/politics/coronavirus-ventilators.html) (2020).
11. New York Times, The U.S. Tried to Build a New Fleet of Ventilators. The Mission Failed. (https://www.nytimes.com/2020/03/29/business/coronavirus-us-ventilator-shortage.html) (2020).
